# Cardiac function is regulated by the sodium-dependent inhibition of the sodium-calcium exchanger NCX1

**DOI:** 10.1038/s41467-024-47850-z

**Published:** 2024-05-07

**Authors:** Kyle Scranton, Scott John, Marina Angelini, Federica Steccanella, Soban Umar, Rui Zhang, Joshua I. Goldhaber, Riccardo Olcese, Michela Ottolia

**Affiliations:** 1 Department of Anesthesiology & Perioperative Medicine, Division of Molecular Medicine, David Geffen School of Medicine, University of California, Los Angeles, Los Angeles, CA USA; 2 Department of Medicine, Division of Cardiology, David Geffen School of Medicine, University of California, Los Angeles, Los Angeles, CA USA; 3 Smidt Heart Institute, Cedars-Sinai Medical Center, Los Angeles, CA USA; 4 Department of Physiology, David Geffen School of Medicine, University of California, Los Angeles, Los Angeles, CA USA

**Keywords:** Cardiovascular biology, Ion transport, Cell biology

## Abstract

The Na^+^-Ca^2+^ exchanger (NCX1) is the dominant Ca^2+^ extrusion mechanism in cardiac myocytes. NCX1 activity is inhibited by intracellular Na^+^ via a process known as Na^+^-dependent inactivation. A central question is whether this inactivation plays a physiological role in heart function. Using CRISPR/Cas9, we inserted the K229Q mutation in the gene (*Slc8a1*) encoding for NCX1. This mutation removes the Na^+^-dependent inactivation while preserving transport properties and other allosteric regulations. NCX1 mRNA levels, protein expression, and protein localization are unchanged in K229Q male mice. However, they exhibit reduced left ventricular ejection fraction and fractional shortening, while displaying a prolonged QT interval. K229Q ventricular myocytes show enhanced NCX1 activity, resulting in action potential prolongation, higher incidence of aberrant action potentials, a faster decline of Ca^2+^ transients, and depressed cell shortening. The results demonstrate that NCX1 Na^+^-dependent inactivation plays an essential role in heart function by affecting both cardiac excitability and contractility.

## Abstract (web summary)

The sodium-calcium exchanger (NCX1) is the primary calcium extrusion mechanism of cardiac myocytes. Here, the authors show that removal of a long questioned allosteric regulation of NCX1 by intracellular sodium alters cardiac excitation-contraction coupling.

## Introduction

Cardiac function relies on the precise quantitative control of both the temporal and spatial distribution of intracellular Ca^2+^. While many proteins contribute to this process, the plasma membrane Na^+^-Ca^2+^ exchanger (NCX1) has been recognized as one of the most influential within this group of Ca^2+^-controlling proteins^1–4^. NCX1 moves Ca^2+^ out of the cell, against its gradient, by harnessing the energy stored in the electrochemical gradient of Na^+^ as a driving force. For each Ca^2+^ ion extruded, three Na^+^ ions are transported into the cell, resulting in the movement of a net positive charge across the sarcolemma. As such, NCX1 can control both heart contractility by modulating Ca^2+^ content and excitability via its electrogenic properties^1,5^. Because of its central role in cardiac function, NCX1 activity is tightly regulated by various cytosolic factors. Among them is intracellular Na^+^ that, in addition to being transported, suppresses NCX1 activity via an intrinsic process known as Na^+^-dependent inactivation^6–9^. This regulatory mechanism manifests as a time-dependent decline of the exchanger current^6,10^. The degree of inactivation is augmented by cytosolic acidification^11–13^, while increases in intracellular Ca^2+^ rescue the exchanger from this state^14–16^.

The physiological relevance of NCX1 Na^+^-dependent inactivation has been debated for more than 30 years, mainly due to the inability of dissecting the role of Na^+^ as either a substrate or regulator when investigated in native tissue. Moreover, electrophysiological studies^6–8^ show that the intracellular Na^+^ concentration must reach 14–18 mM to inactivate ~half of the exchanger population. Such Na^+^ concentrations are considered above the physiological range^17^, further undermining the potential role of this regulation in vivo. However, Na^+^ levels sensed by NCX1 may be higher due to its proximity to Na^+^ channels^18^ and the presence of subsarcolemmal Na^+^ gradients^19^. Also, cytosolic Na^+^ concentrations above 20 mM have been associated with pathological conditions such as ischemia/reperfusion and chronic heart failure^20,21^. These scenarios may allow this regulatory mechanism to develop in vivo. Finally, Na^+^-dependent inactivation is conserved across most members of the Na^+^-Ca^2+^ exchanger family^1,22,23^, manifesting in various cell types^8,12,24,25^, including cardiac myocytes^6,7^, suggesting an evolutionary pressure to maintain such regulation.

Based on these premises, we sought to determine whether the Na^+^-dependent inactivation plays a role in cardiac physiology, by creating a new mouse line in which the native exchanger (NCX1) was genetically manipulated to lack this regulation. This was accomplished by replacing the lysine at position 229 with a glutamine via the CRISPR/Cas9 technique^26^. This substitution exclusively removes the Na^+^-dependent inactivation without altering NCX1 affinity for the transported ions or other regulatory properties^8,13^. Such a strategy has allowed us to provide experimental evidence that NCX1 Na^+^-dependent inactivation is an important component of cardiac function as its ablation alters heart contractility and excitability in physiological settings.

It is well established that Na^+^ is a key player in cardiac physiology^17,20^ and our study reveals a new mechanism by which this ion exerts this essential task. This Na^+^-dependent signaling pathway may have important clinical implications considering that elevations in intracellular Na^+^ are linked to cardiac pathologies such as heart failure and ischemia/reperfusion injury^17,21,27^.

## Results

### Genetic ablation of NCX1 Na^+^-dependent inactivation results in viable mice

**Fig. 1:**
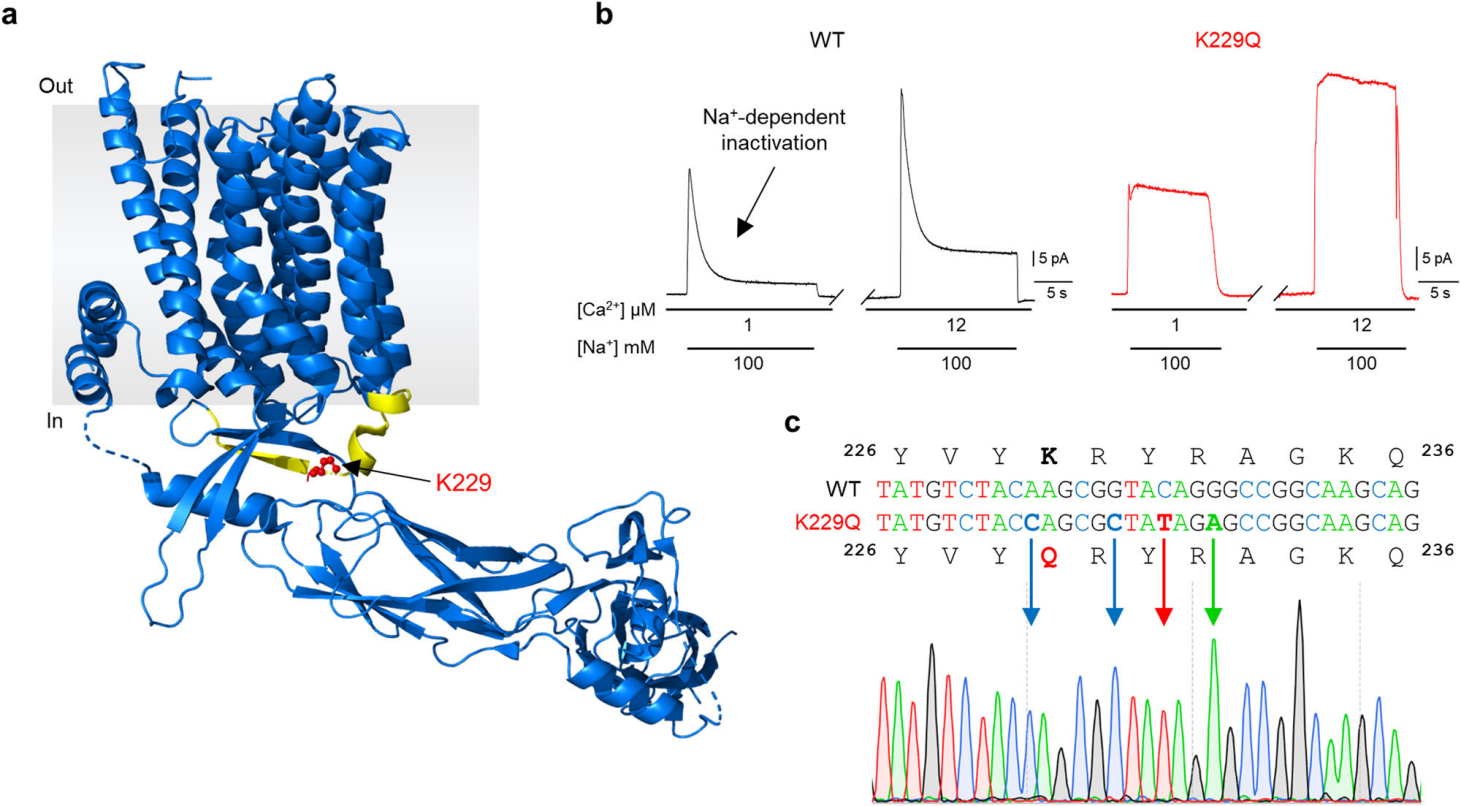
NCX1 Na^+^-dependent inactivation is removed via K229Q mutation. **a** Structure of human cardiac NCX1 modified from PDB: 8SGJ^28^. The XIP region, aa 219–238, is highlighted in yellow. Lysine 229 (red) is located within the XIP region and was replaced with glutamine, establishing NCX1 K229Q. Numbering excludes the signal peptide. **b** Representative outward currents recorded from the mouse cardiac exchanger expressed in *Xenopus laevis* oocytes. To elicit NCX1 outward current, cytoplasmic Cs^+^ (100 mM, not transported) was rapidly replaced with Na^+^ (100 mM) in the presence of the indicated cytosolic Ca^2+^ concentrations (determined via Ca^2+^ electrode measurement). Lines below the traces indicate solution changes. WT currents (black) peaked and then decayed over several seconds due to Na^+^-dependent inactivation. Increasing the cytoplasmic Ca^2+^ content enhanced the WT current and decreased the percentage of inactivated exchangers. In contrast, K229Q mutants (red) showed sustained NCX1 outward current in the presence of 100 mM Na^+^, indicating the removal of Na^+^-dependent inactivation while preserving its sensitivity to intracellular Ca^2+^ (as previously been shown in the canine NCX1^8^). Experiments were conducted at a holding potential of 0 mV and at 35 °C. **c** Mutation K229Q was inserted into the coding region of the NCX1 gene (*Slc8a1*) via CRISPR/Cas9, generating the mouse line K229Q. DNA sequences of mouse NCX1 WT (top) and designed K229Q mutant (bottom) with chromatogram showing homozygosity of K229Q mutation.

Millimolar concentrations of cytosolic Na^+^ inhibit NCX1 transport by driving the protein into an inactivated state^6,8^. Previous investigations have implicated a stretch of conserved residues, referred to as the XIP region (aa 219–238, Fig. 1a)^1,28^, in this process known as Na^+^-dependent inactivation, and shown that replacement of lysine 229 to glutamine within XIP abolishes this regulation without altering other properties of NCX1^8,29,30^.

We confirmed these findings by expressing the mouse cardiac wild-type (NCX1.1, WT) or K229Q mutant exchanger in the *Xenopus* oocyte expression system. Outward exchanger currents were recorded using the giant patch technique in the excised inside-out configuration^13^. As shown in Fig. 1b, WT ionic current peaks and then decays to a steady state value due to the Na^+^-dependent inactivation process^6^. Increases in intracellular Ca^2+^ concentration augmented the exchanger current and decreased the extent of inactivation^14^. In contrast, the K229Q outward current did not display the Na^+^-dependent decay while maintaining a similar response to changes in intracellular Ca^2+^ as the WT exchanger. The results confirm previous reports showing that the K229Q mutation does not alter other allosteric modulations of NCX1 or its transport properties^8,13^. Upon this foundation, the K229Q mutation was introduced into the mouse *Slc8a1* gene via CRISPR/Cas9 (Fig. 1c), generating a mouse line named K229Q. The *Slc8a1* gene encodes for NCX1^1^, including the only known cardiac splice isoform, NCX1.1^1,31^ (indicated as NCX1 throughout the manuscript).

**Fig. 2:**
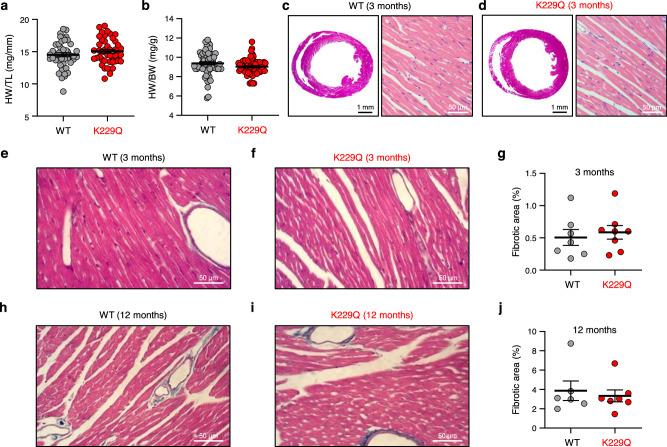
Removal of Na^+^-dependent inactivation does not affect heart size or fibrosis development. **a**, **b** No significant difference in heart weight (HW) to tibia length (TL) or body weight (BW) ratio was determined in WT *vs* K229Q male mice (HW/TL, WT, gray, *n* = 45; K229Q, red, *n* = 44) (HW/BW, WT, gray, *n* = 61; K229Q, red, *n* = 58). **c**, **d** Representative H&E staining of paraffin-embedded WT and K229Q 12–16 week old (3 months) heart sections showing comparable heart size between the two groups. The results indicate that ablation of NCX1 Na^+^-dependent inactivation does not lead to a significant change in heart size. **e**, **f**, **h**, **i** Representative images of paraffin-embedded, Masson’s Trichrome stained heart sections of WT and K229Q mice collected at 12–16 weeks (3 months) (**e**, **f**) and 50–54 weeks (12 months) (**h**, **i**) of age. **g**, **j** The percentage of total fibrosis was calculated as the summed blue stained area divided by the total area. Results show no difference in fibrosis content between WT and K229Q mice (12–16 weeks: WT, gray, *n* = 7; K229Q, red, *n* = 8) (50–54 weeks: WT, gray, n = 6; K229Q, red, *n* = 7). All data reported as mean ± SEM (two-tailed Welch’s *t*-test). Source data are provided in the Source Data file.

The homozygous K229Q mice are viable and fertile, living to adulthood with no readily discernible behavioral or physical phenotypes, indicating that the Na^+^-dependent inactivation of NCX1 plays little role in essential developmental processes under normal animal husbandry conditions. Consistent with this observation, we measured similar heart weight to tibia length and heart weight to body weight ratios between WT and K229Q mice (Fig. 2a, b). Hematoxylin and eosin (H&E) staining of ventricular tissue cross-sections revealed similar cardiac anatomy and morphology between WT and K229Q mice at 3 months (Fig. 2c, d). Finally, there was no significant difference in total fibrotic area between WT and K229Q ventricular tissue cross-sections at 12–16 weeks of age (3 months) (WT = 0.51 ± 0.12%, *n* = 7; K229Q = 0.59 ± 0.10%, *n* = 8; *P* = 0.634), as assessed by Masson’s trichrome staining (Fig. 2e–g). This result was confirmed in aged mice (12 months, Fig. 2h–j) which showed similar increases in fibrotic content for both WT and K229Q mice (WT = 3.87 ± 1.01%, *n* = 6; K229Q = 3.34 ± 0.61%, *n* = 7; *P* = 0.664).

### NCX1 inhibition by cytosolic Na^+^ controls cardiac function in live animals

**Fig. 3:**
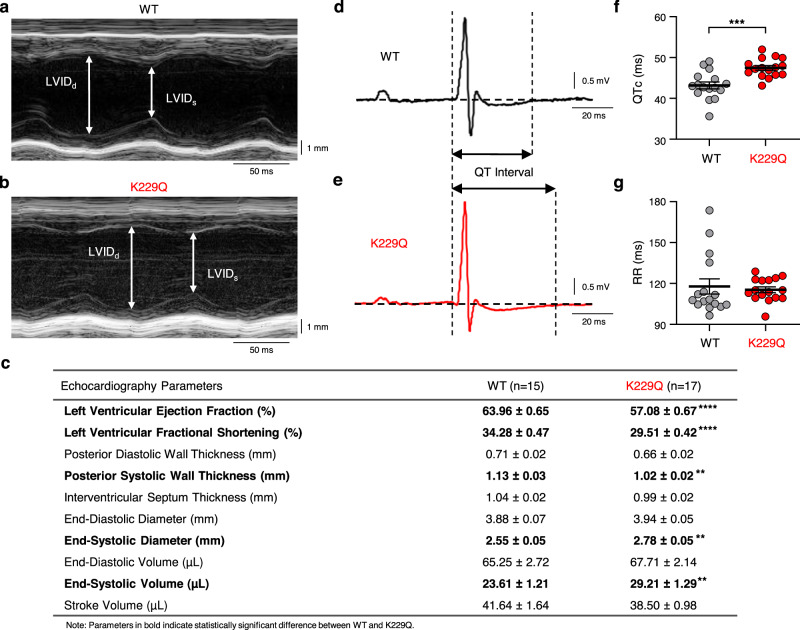
Na^+^-dependent inactivation modulates cardiac function in live animals. **a**, **b** Heart properties were evaluated via echocardiography in lightly anesthetized mice and shown are representative M-mode images of 3-month-old WT (**a**) and K229Q mice (**b**). **c** Summary of the echocardiography parameters. Significantly different parameters are highlighted in bold. Left ventricular ejection fraction and fractional shortening is decreased in K229Q mice, indicating that NCX1 Na^+^-dependent inactivation modulates cardiac contractility. The number of animals used for the investigations are shown in the column heading. **d**, **e** Representative surface ECG recordings were taken from lightly anesthetized WT (**d**, black) and K229Q (**e**, red) mice. The QT interval is highlighted via vertical dashed lines. K229Q mice show prolonged QT interval. **f** Summary data comparing the duration of the heart rate-corrected QT intervals (QTc) (Bazett’s formula)^75^ recorded from WT and K229Q mice. QTc intervals were significantly longer in K229Q mice (WT, gray, *n* = 16; K229Q, red, *n* = 16). Similar results were observed for non-corrected QT values (WT = 46.57 ± 1.21 ms, *n* = 16; K229Q = 50.97 ± 0.86 ms, *n* = 16; *P* = 0.006). **g** Summary data for the RR interval recorded from WT and K229Q mice (WT, gray, *n* = 16; K229Q, red, *n* = 16; *P* = 0.692). Values were not significantly different. All values are reported as the mean ± SEM (two-tailed Welch’s *t*-test; ****P < 0.0001, ***P < 0.001, **P < 0.01). Specific *P* values and source data are provided in the Source Data file.

To assess the effects of NCX1 Na^+^-dependent inactivation on the physiological properties of the heart, echocardiography and surface electrocardiograms (ECG) were conducted in lightly sedated animals. Echocardiography measurements obtained at 12–16 weeks of age (Fig. 3a–c) depict a modest decrease in the systolic left ventricle (LV) wall thickness in the K229Q mice, while the interventricular septum thickness was unchanged between WT and K229Q mice (Fig. 3c). The results indicate that removal of the exchanger Na^+^-dependent inactivation does not induce hypertrophy. Notably, K229Q mouse hearts displayed a decrease in left ventricular ejection fraction (EF: WT = 63.96 ± 0.65% *n* = 15; K229Q = 57.08 ± 0.67%, *n* = 17; *P* < 0.0001) and fractional shortening when compared to WT mice (FS: WT = 34.28 ± 0.47%, *n* = 15; K229Q = 29.51 ± 0.42%, *n* = 17; *P* < 0.0001). Consistent with the impaired contractility, the LV end-systolic diameter and LV systolic volume (Fig. 3c) were augmented in the K229Q mice. These differences became more pronounced with age (Supplementary Fig. 1), suggesting that the absence of Na^+^-dependent inactivation progressively stresses the heart even though fibrotic changes were not readily apparent (Fig. 2h–j).

### Surface ECG of K229Q mice displayed prolonged QT interval

The observed changes in contractility were accompanied by altered electrical properties in the hearts of K229Q animals. Analysis of surface ECG recordings (Fig. 3d–f) revealed a significant prolongation of the QT interval recorded from K229Q mice (corrected QT, QTc: WT = 43.17 ± 0.84 ms, *n* = 16; K229Q = 47.46 ± 0.57 ms, *n* = 16; *P* = 0.0003), while the RR interval did not differ between the WT and K229Q mice (Fig. 3g) (RR interval: WT = 117.8 ± 5.5 ms, *n* = 16; K229Q = 115.4 ± 2.1 ms, *n* = 16; *P* = 0.692).

### K229Q myocytes displayed prolonged action potential duration

**Fig. 4:**
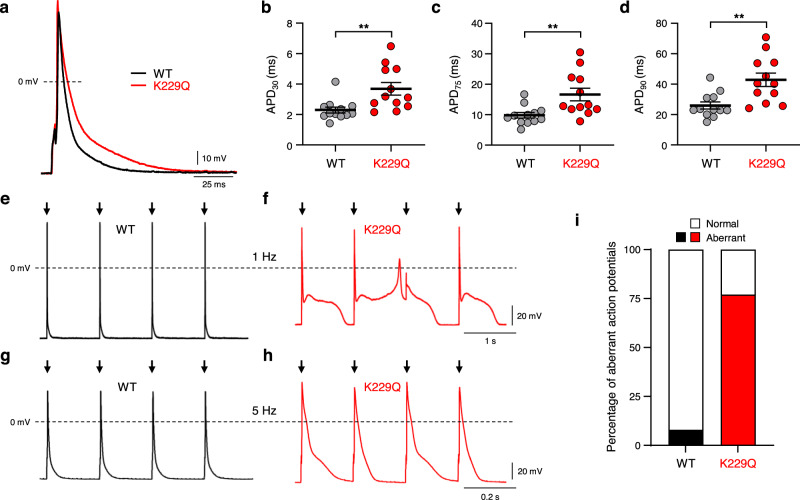
Ablation of NCX1 Na^+^-dependent inactivation prolongs action potential duration. **a** Representative action potential recordings from WT (black) and K229Q (red) adult ventricular myocytes. K229Q myocytes displayed prolonged action potential duration when compared to WT cells. **b**–**d** Action potential duration (APD) was measured at 30%, 75%, and 90% repolarization (WT, gray, *n* = 12/8 cells/animals; K229Q, red, *n* = 12/6 cells/animals; 1 Hz). **e**–**h** Shown are action potentials recorded from WT (**e**, **g**, black) and K229Q (**f**, **h**, red) ventricular myocytes paced at the indicated frequency. K229Q myocytes were more prone to elicit aberrant AP displaying early afterdepolarizations (EADs) (**f**) and action potential alternans (**h**). **i** The graph summarizes the fraction of normal and aberrant action potentials in WT and K229Q myocytes. White indicates the percentage of normal action potentials, while black/red indicates the percentage of aberrant action potentials (including both EADs and alternans) for WT and K229Q, respectively (aberrant cells/total cells: WT *n* = 1/12; K229Q *n* = 10/13). Data reported as the mean ± SEM (two-tailed Welch’s *t*-test; **P < 0.01). Source data are provided in the Source Data file.

The ECG recordings from lightly sedated animals (Fig. 3d–f) show that K229Q mice display a prolonged QT interval, indicating a prolonged action potential duration (APD) at the single-cell level. Accordingly, we compared action potentials recorded from isolated WT and K229Q adult ventricular myocytes using the patch-clamp technique in the current-clamp mode. We found that K229Q myocytes have a significantly prolonged action potential (Fig. 4a–d) (APD_90_: WT = 25.99 ± 2.36 ms, *n* = 12/8 cells/animals; K229Q = 42.91 ± 4.39 ms, *n* = 12/6 cells/animals; *P* = 0.004). The resting membrane potential was unaltered between the two groups (WT = −68.16 ± 0.61 mV, *n* = 12/8 cells/animals; K229Q = −68.29 ± 1.28 mV, *n* = 12/6 cells/animals; *P* = 0.928).

Myocytes isolated from K229Q mice showed a higher incidence of aberrant action potentials. Prolonged action potentials with failure to repolarize, resembling early afterdepolarizations (EADs) (Fig. 4f), were observed in 4 out of 13 K229Q homozygous myocytes and 8 out of 11 myocytes expressing the mutant exchanger showed beat-to-beat alternans in action potential duration when paced at 5 Hz (Fig. 4h). The fraction of cells presenting aberrant action potentials in WT and K229Q is summarized in Fig. 4i (*P* = 0.001 via Fisher’s exact test).

### K229Q mutation enhances NCX1 current in cardiac myocytes

**Fig. 5:**
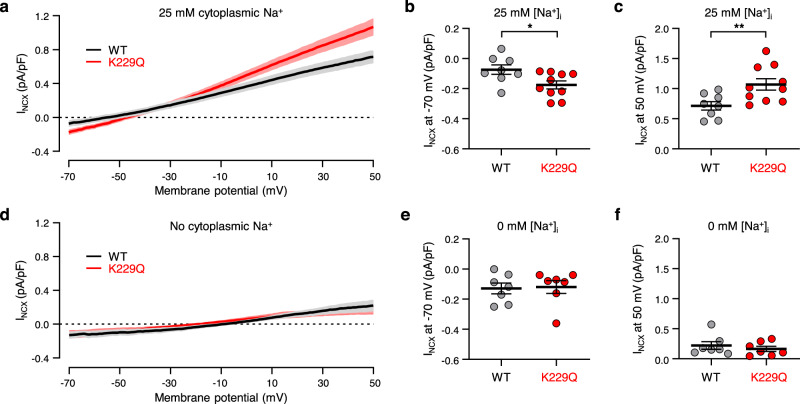
NCX1 currents recorded from K229Q myocytes are enhanced due to the removal of Na^+^-dependent inactivation. **a** Summary of nickel-sensitive I_NCX_ currents recorded from WT (black) and K229Q (red) adult ventricular myocytes. Whole-cell NCX1 currents (I_NCX_) were elicited using a ramp protocol in the presence of 25 mM intracellular Na^+^. I_NCX_ was defined as the nickel-sensitive component determined by subtracting nickel-insensitive current from the total current. Bold lines represent the averaged nickel-sensitive I_NCX_ currents normalized to cell capacitance, while lighter shading represents SEM (WT *n* = 8/4 cells/animals; K229Q *n* = 10/6 cells/animals). **b**, **c** Shown are the average values of the inward (**b**) and outward (**c**) WT (gray) and K229Q (red) NCX1 current densities, measured at the indicated voltage. The results show that K229Q exchangers elicit enhanced currents, as they do not undergo Na^+^-dependent inactivation. **d** Nickel-sensitive I_NCX_ currents recorded from WT (black) and K229Q (red) myocytes in the absence of intracellular Na^+^ (WT *n* = 7/5 cells/animals; K229Q *n* = 7/3 cells/animals). **e**, **f** I_NCX_ current densities were measured at the indicated voltage membrane potential. The graphs demonstrate that the WT (gray) and K229Q (red) exchangers elicit similar current amplitudes in ionic conditions that do not allow for Na^+^-dependent inactivation (0 mM intracellular Na^+^). All values are reported as the mean ± SEM (two-tailed Welch’s *t*-test; **P < 0.01, *P = 0.05). Experiments were conducted at 33 °C. Specific *P* values and source data are provided in the Source Data file.

The data described above provide experimental evidence that the Na^+^-dependent inactivation of NCX1 shapes the electrical properties of the heart by prolonging the cardiac action potential and thus the QT interval. To investigate the mechanisms leading to these altered properties, we first determined the effects of the K229Q mutation on NCX1 current in isolated ventricular myocytes. NCX1 activity was recorded from WT and K229Q adult ventricular myocytes in the presence of 25 mM intracellular Na^+^. The elevated intracellular Na^+^ will drive WT exchangers into the inactivated state while it will be ineffective on the K229Q mutant. Accordingly, NCX1 currents recorded from K229Q myocytes should be enhanced. As expected, K229Q myocytes displayed a significant enhancement in current amplitude when compared to WT cells (Fig. 5a–c) (Inward I_NCX_ currents at −70 mV: WT = −0.07 ± 0.03 pA/pF, *n* = 8/4 cells/animals; K229Q = −0.18 ± 0.03 pA/pF, *n* = 10/6 cells/animals; *P* = 0.026; Outward I_NCX_ currents at 50 mV: WT = 0.72 ± 0.07 pA/pF; K229Q = 1.07 ± 0.10 pA/pF, *P* = 0.009). The result is consistent with the increased activity of the exchanger carrying mutation K229Q due to the absence of Na^+^-dependent inactivation. The enhanced Na^+^ influx via the mutated exchanger may contribute to the delayed repolarizations seen in K229Q myocytes (Fig. 4).

Next, we examined whether mutation K229Q alters NCX1 biophysical properties other than removing the Na^+^-dependent inactivation. For this purpose, we compared WT and K229Q exchanger ionic currents recorded from adult ventricular myocytes in the absence of intracellular Na^+^. The absence of cytosolic Na^+^ will prevent the WT exchanger from occupying the inactivated state and thus, WT and K229Q exchangers should display comparable activity when measured in this ionic condition. As shown in Fig. 5d–f, WT and K229Q inward (forward mode) and outward (reverse mode) currents had the same amplitude (Inward I_NCX_ currents at −70 mV: WT = −0.13 ± 0.04 pA/pF, *n* = 7/5 cells/animals; K229Q = −0.12 ± 0.04 pA/pF, *n* = 7/3 cells/animals, *P* = 0.87; Outward I_NCX_ currents at 50 mV: WT = 0.22 ± 0.06 pA/pF; K229Q = 0.16 ± 0.04 pA/pF, *P* = 0.48) confirming that, in the absence of intracellular Na^+^, WT and K229Q exchangers display the same transport properties, supporting the view that mutation K229Q does not elicit secondary effects on NCX1 transport activity.

### The K229Q mutation does not affect NCX1 expression or membrane trafficking

**Fig. 6:**
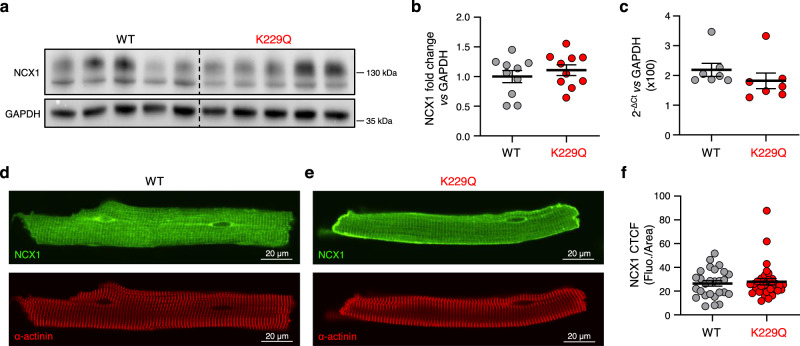
Mutation K229Q does not alter NCX1 expression and trafficking in cardiac myocytes. **a** Representative Western blots for NCX1 and GAPDH, which served as a loading control. Each lane represents a lysate from WT or K229Q ventricular tissue. **b** NCX1 protein levels were quantified via chemiluminescence of HRP-bound protein and values were normalized to the corresponding loading control (GAPDH) (WT, gray; K229Q, red; *n* = 10 animals per group). **c** Shown are NCX1 transcript levels obtained from WT (gray) and K229Q (red) adult ventricular myocytes (*n* = 7 animals per group). The mRNA levels of WT and mutant K229Q exchangers were equivalent. **d**, **e** Immunofluorescence images of adult ventricular myocytes isolated from WT (**d**) and K229Q (**e**) hearts. Cells were fixed and stained with anti-NCX1 (RF31, green) and anti-α-actinin (red) antibodies and imaged with confocal microscopy. **f** NCX1 fluorescence levels were quantified by determining the corrected total cell fluorescence (CTCF), as detailed in the methods (WT, gray, *n* = 27/4 cells/animals; K229Q, red, *n* = 31/4 cells/animals). The results are the mean ± SEM. The data confirm previous reports that the K229Q mutation does not alter NCX1 expression nor membrane localization^8,24^. Source data are provided in the Source Data file.

To rule out the possibility that the enhanced NCX1 currents recorded from K229Q myocytes arise from increased levels of expression of the mutant exchanger, we performed immunoblot analysis. As shown (Fig. 6a, b), the amount of NCX1 protein was comparable between the WT and K229Q ventricular tissues. Consistent with this result, RT-qPCR demonstrated equivalent amounts of mRNA encoding for WT and K229Q exchangers (Fig. 6c). The mRNA levels of the two additional NCX isoforms, NCX2 and NCX3, remained undetectable in both WT and mutant myocytes (Supplementary Fig. 2a), indicating that removal of NCX1 Na^+^ modulation does not induce isoform switching or enhancement.

Next, we determined if the K229Q mutation altered NCX1 trafficking in cardiac myocytes by examining the localization of the WT and mutated exchanger using fluorescent immunocytochemistry. Adult ventricular myocytes were immunostained using a monoclonal antibody against the cardiac Na^+^-Ca^2+^ exchanger (RF31)^32^. As shown in Fig. 6, mutation K229Q did not alter NCX1 distribution in the sarcolemma or T-tubules of adult ventricular myocytes (Fig. 6d–f). K229Q myocytes also displayed comparable staining for sarcomeric α-actinin distribution (Fig. 6d, e), indicating unchanged structural organization.

Finally, we evaluated the mRNA levels of other key proteins involved in excitation-contraction (EC) coupling. RT-qPCR studies demonstrated that the transcript levels of the voltage-dependent Na^+^ (Na_V_1.5, *Scn5a*), Ca^2+^ (Ca_V_1.2, *Cacna1c*), and K^+^ (K_V_4.2, *Kcnd2*) channels in cardiac myocytes expressing the K229Q exchanger were comparable to those measured in WT myocytes (Supplementary Fig. 2a). Similar results were obtained for the Na^+^/K^+^-ATPase (NKA, *Atp1a1*), the sarcoplasmic Ca^2+^-ATPase (SERCA, *Atp2a2*), the ryanodine receptor (RYR2, *Ryr2*), and Ca^2+^/calmodulin dependent protein kinases II delta/gamma (CaMK2D, *Camk2d*; CaMK2G, *Camk2g*) (Supplementary Fig. 2b–e). Taken together, these findings provide evidence that the altered cardiac function observed in K229Q mice is not due to changes in the expression level or pattern of expression of NCX1, nor the transcript levels of other proteins associated with EC coupling.

### L-type Ca^2+^ currents are not altered in K229Q myocytes

**Fig. 7:**
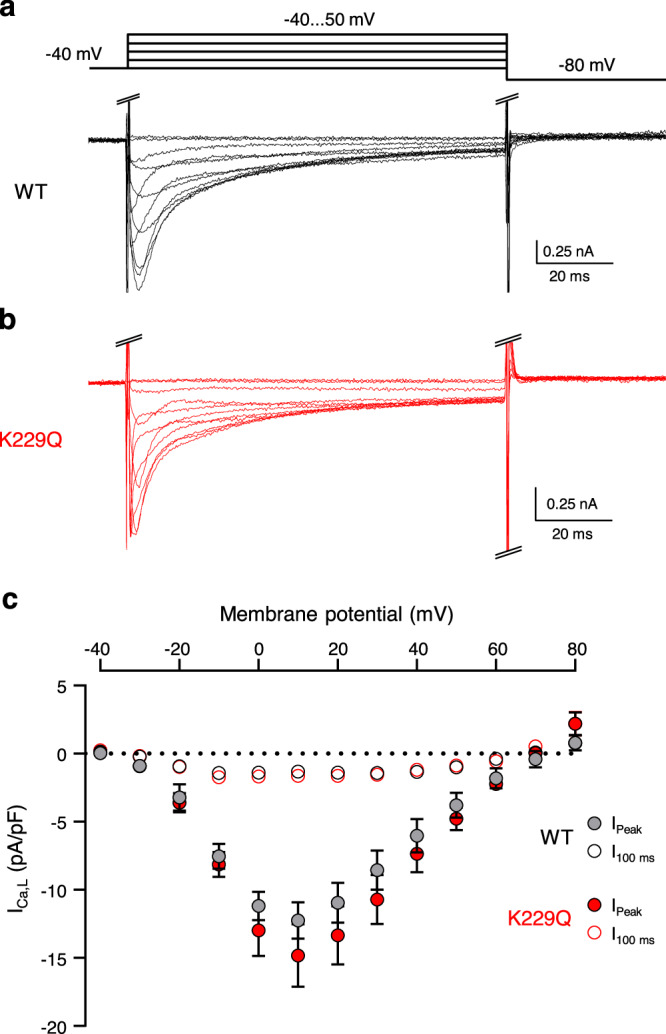
WT and K229Q myocytes display comparable L-type Ca^2+^ current (I_Ca,L_) density. **a**, **b** Representative nifedipine-sensitive L-type Ca^2+^ current (I_Ca,L_) recordings measured from WT (**a**, black) and K229Q (**b**, red) adult ventricular myocytes. Currents were elicited using the protocol shown above traces. I_Ca,L_ were obtained by applying nifedipine (0.02 mM) at the end of the experiments and subtracting nifedipine-insensitive current from the total current. The resulting currents were then normalized to cell capacitance. Experiments were conducted at 35 °C. **c** Summary of the current-voltage relationship of WT (gray) and K229Q (red) I_Ca,L_. Filled symbols represent the peak currents values (I_Peak_) while empty symbols refer to the late current (I_100ms_) measured at the end of the depolarizing pulse. Each point is the average of 7 cells for WT (5 animals) and 6 cells for K229Q (3 animals). No significant difference in Ca^2+^ current density was observed between WT and K229Q myocytes. Data reported as the mean ± SEM (two-tailed Welch’s *t*-test). Source data are provided in the Source Data file.

As Ca^2+^ influx via the L-type Ca^2+^ channels is an important contributor to membrane excitability and was found to be altered in transgenic NCX1 knock-out mice^33,34^, we directly examined L-type Ca^2+^ current density in WT and K229Q myocytes. Representative nifedipine-sensitive current recordings are shown in Fig. 7. The peak (I_Peak_) and the late non-inactivating component (I_100 ms_) of the Ca^2+^ currents were similar in the WT and K229Q myocytes. The result indicates that this depolarizing current does not contribute to action potential prolongation seen in K229Q myocytes and that mutation K229Q does not elicit secondary adaptations leading to altered Ca^2+^ channel currents. This is consistent with reports showing that overexpression of NCX1 resulted in the prolongation of the action potential without enhancing Ca^2+^ currents^35,36^.

### Suppression of NCX1 Na^+^-dependent inactivation accelerates Ca^2+^ transient decay and decreases cell shortening

**Fig. 8:**
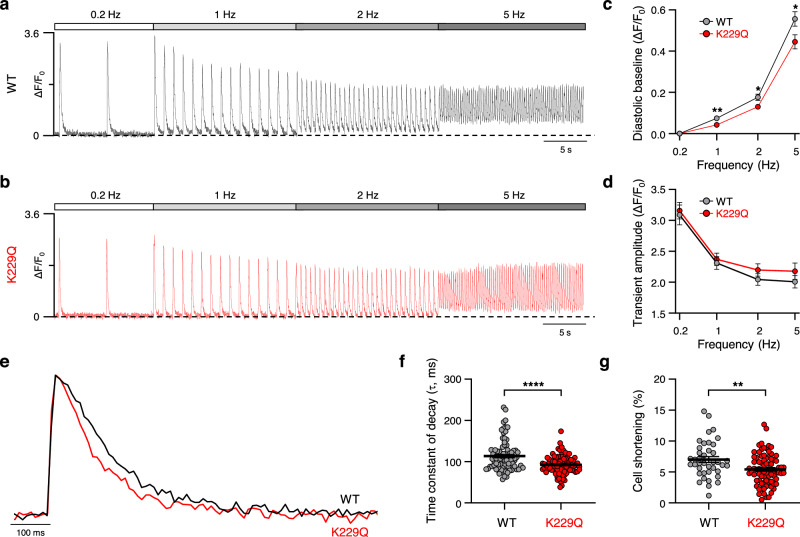
Frequency dependence of Ca^2+^ transients in WT and K229Q adult ventricular myocytes. **a**, **b** Representative Ca^2+^ transients recorded from isolated WT (**a**, black) and K229Q (**b**, red) adult ventricular myocytes. Myocytes were externally paced at the indicated frequencies. ΔF/F_0_ was calculated using the baseline fluorescence at 0.2 Hz as F_0_. **c** The plot depicts how diastolic fluorescence changes with increases in pacing rate in WT and K229Q myocytes (WT, gray, *n* = 65/10 cells/animals for 0.2–2 Hz, *n* = 40/5 cells/animals for 5 Hz; K229Q, red, *n* = 52/9 cells/animals for 0.2–2 Hz and *n* = 37/6 cells/animals for 5 Hz). The increase in baseline Ca^2+^ levels at higher frequencies was less pronounced in K229Q myocytes than in WT cells. The result suggests enhanced Ca^2+^ extrusion by the mutated exchanger. If not visible, error bars are within the symbol. **d** Summary data of Ca^2+^ transient amplitude *vs*. stimulation frequency for WT and K229Q myocytes. WT and K229Q myocytes displayed similar Ca^2+^ transient amplitude at all frequencies (WT, gray, *n* = 65/10 cells/animals for 0.2–2 Hz, *n* = 40/5 cells/animals for 5 Hz; K229Q, red, *n* = 52/9 cells/animals for 0.2–2 Hz and *n* = 37/6 cells/animals for 5 Hz). **e** Shown are representative Ca^2+^ transients recorded at 1 Hz from WT (black) and K229Q (red) adult ventricular myocytes. Traces were normalized to the peak and superimposed. Results show that K229Q myocytes have Ca^2+^ transients with a faster decay than those measured from WT cells. **f** Summary data for the WT and K229Q time constant (τ) of Ca^2+^ transient decay. Removal of NCX1 Na^+^-dependent inactivation increases Ca^2+^ extrusion resulting in a significant decrease in the time constant (WT, gray, *n* = 85/16 cells/animals; K229Q, red, *n* = 89/16 cells/animals). **g** Contractility of adult ventricular myocytes was assessed as cell shortening measured from cells paced at 1 Hz and expressed as the percentage of resting cell length. K229Q myocytes showed decreased unloaded cell shortening (WT, gray, *n* = 40/6 cells/animals; K229Q, red, *n* = 80/8 cells/animals). Data are shown as the mean ± SEM (two-tailed Welch’s *t*-test; ^****^P < 0.0001, ^**^P < 0.01, ^*^P < 0.05). Source data and specific *P* values are provided in the Source Data file.

The echocardiography data shown in Fig. 3c indicate reduced contractility in K229Q animals, suggesting alterations in the process of excitation-contraction coupling. To investigate whether the removal of the Na^+^-dependent inactivation impairs Ca^2+^ dynamics, we compared Ca^2+^ transients recorded from isolated WT and K229Q ventricular myocytes loaded with the Ca^2+^-sensitive dye Fluo-4 AM. Cells were challenged with increasing pacing frequencies as high stimulation rates are associated with elevation in both Ca^2+^ and Na^+^ levels as well as a concomitant decrease in Ca^2+^ transient amplitude^37,38^. Examples of Ca^2+^ transients recorded from myocytes paced at increasing frequencies are shown in Fig. 8. As expected, WT myocytes showed an increase in the baseline fluorescence with increased pacing, reflecting the associated elevation in diastolic Ca^2+^^38^ (Fig. 8a, c). Notably, the enhancement of baseline fluorescence was significantly less in K229Q myocytes challenged with the same protocol (Fig. 8b, c). On average, there were no significant changes in transient amplitude between WT and K229Q myocytes at all frequencies (Fig. 8d).

The smaller increase in baseline fluorescence seen in K229Q myocytes during changes in the rate of stimulation suggests enhanced exchanger activity due to the absence of Na^+^-dependent inactivation, leading to increased Ca^2+^ efflux. Consistently, the time constant of the Ca^2+^ transient decay was reduced by ~19% in K229Q myocytes (Fig. 8e, f) (τ values: WT = 113.50 ± 3.97 ms, *n* = 85/16 cells/animals; K229Q = 92.37 ± 2.36 ms, *n* = 89/16 cells/animals; 1 Hz, *P* < 0.0001), resulting in transients of shorter duration (WT = 122.8 ± 2.4 ms, *n* = 85/16 cells/animals; K229Q = 104.3 ± 1.3 ms, *n* = 89/16 cells/animals; 1 Hz, *P* < 0.0001, measured at half maximum). In addition to the accelerated Ca^2+^ transient decay, K229Q myocytes displayed a significant decrease in unloaded cell shortening when expressed as a percentage of resting cell length (WT = 7.0 ± 0.5%, *n* = 40/6 cells/animals; K229Q = 5.4 ± 0.3%, *n* = 80/8 cells/animals; *P* = 0.005) (Fig. 8g), a finding that is consistent with the observed reduction in ejection fraction (Fig. 3c).

### WT and K229Q myocytes have similar sarcoplasmic reticulum Ca^2+^ content and SERCA activity

**Fig. 9:**
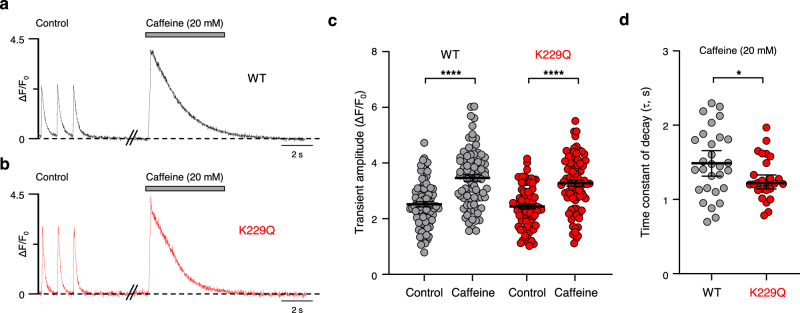
K229Q myocytes show unaltered SR Ca^2+^ content. **a**, **b** Representative control and caffeine-induced Ca^2+^ transients recorded from WT (**a**, black) and K229Q (**b**, red) adult ventricular myocytes. After equilibration of the SR content (pacing at 1 Hz for 60 s), myocytes were paced at 1 Hz for 15 s to record control transients. Only the last three transients are shown. Stimulation was then stopped and, after a 4 s delay, 20 mM caffeine was applied for 5 s. **c** Summary data showing the Ca^2+^ transient amplitude (ΔF/F_0_) during control and after caffeine application. Results indicate that removal of Na^+^-dependent inactivation does not alter Ca^2+^ transient amplitudes induced by caffeine, indicating that Ca^2+^ released from SR is unchanged in K229Q myocytes (WT, gray, *n* = 81/15 cells/animals; K229Q, red, *n* = 81/11 cells/animals). **d** Time constant of decay (τ) of caffeine-induced Ca^2+^ transients recorded from WT and K229Q myocytes. K229Q myocyte time constant of decay is decreased when compared to the τ value measured from WT cells (WT, gray, *n* = 29/6 cells/animals; K229Q, red, *n* = 26/7). Tau was determined by single exponential fitting. All values are represented as the mean ± SEM (two-tailed Welch’s *t*-test; ****P < 0.0001, *P < 0.05). Specific *P* values and source data are provided in the Source Data file.

The impaired contractility observed in K229Q mice may arise from altered Ca^2+^ reuptake into the sarcoplasmic reticulum (SR). To probe this possibility we compared the amplitude of the WT and K229Q caffeine-induced Ca^2+^ transients as indicators of SR Ca^2+^ load. Adult ventricular myocytes isolated from WT and K229Q mice were loaded with the calcium ion indicator Fluo-4 AM and subjected to electrical field stimulation. To equilibrate the SR’s Ca^2+^ content, myocytes were paced at 1 Hz for 60 s. Subsequently, control transients were recorded followed by a puff of caffeine (20 mM), applied adjacent to the myocytes. Representative Ca^2+^ transients recorded from WT and K229Q myocytes prior to and during caffeine application are shown in Fig. 9a, b. The caffeine-induced Ca^2+^ transients recorded from K229Q myocytes showed comparable amplitude (Fig. 9c) to those measured from WT cells but decayed more rapidly (Fig. 9d) (τ values: WT = 1.49 ± 0.08 s, *n* = 29/6 cells/animals; K229Q = 1.28 ± 0.06 s, *n* = 26/7 cells/animals; *P* = 0.045), suggesting enhanced Ca^2+^ extrusion via K229Q exchanger.

To confirm that the enhanced activity of the K229Q exchanger is responsible for the accelerated decline of the Ca^2+^ transients, we probed SERCA activity in WT and K229Q ventricular myocytes. Myocytes were field stimulated while bathed in normal Tyrode’s solution (1 mM Ca^2+^) to record Ca^2+^ transients under control conditions. The pacing was then paused and cells were perfused for 9 min in a modified Tyrode’s solution lacking both Na^+^ (replaced with equal amounts of Li^+^) and Ca^2+^. Such maneuver depletes the cells of intracellular Na^+^, thereby preventing NCX1 from working in reverse mode (Ca^2+^ influx) upon reintroduction of extracellular Ca^2+^^39^. Myocytes were then reintroduced to Ca^2+^ by switching to a solution containing 1 mM Ca^2+^ and 140 mM Li^+^. As Li^+^ cannot be transported by NCX1, the exchanger is also prevented from operating in the forward mode (Ca^2+^ efflux). Under these conditions, the decay of the Ca^2+^ transient is largely driven by SERCA. Representative WT and K229Q Ca^2+^ transients recorded using this protocol are shown in Supplementary Fig. 3. As expected, K229Q myocytes displayed faster Ca^2+^ transient decay than WT cells when recorded in control conditions (regular Tyrode’s solution, Supplementary Fig. 3c, e). In contrast, preventing NCX1 activity by removing intracellular and extracellular Na^+^ abolished this difference (0 Na^+^, 1 mM Ca^2+^, Supplementary Fig. 3d, f). These findings suggest that SERCA activity is not altered in K229Q myocytes, a result in agreement with the evidence that the amplitude of the caffeine-induced Ca^2+^ transient is not significantly different between WT and K229Q myocytes (Fig. 9c). The data support the conclusion that the acceleration in the Ca^2+^ transient relaxation seen in K229Q myocytes is due to increased NCX1 activity in the absence of Na^+^-dependent inactivation.

## Discussion

The overarching goal of this study was to investigate whether the Na^+^-dependent inactivation of NCX1 is physiologically relevant and necessary for normal cardiac function. Na^+^ is an essential regulator of cardiac function^17,20,27^, but the molecular mechanisms by which it directly influences intracellular Ca^2+^ levels are often overlooked. By inactivating NCX1, changes in intracellular Na^+^ levels could in principle affect both cardiac contractility and excitability. This scenario, however, has been long debated^4,17,20,24,40–43^ primarily because the levels of Na^+^ required to inactivate NCX1 are considered non-physiological. To date, there is no evidence for or against this view because of the difficulties in dissecting out the role of Na^+^ as an inhibitor or transport substrate in native tissues. Thus, this process is often ignored and excluded from current mathematical models of the cardiac action potential^44–47^. The findings herein demonstrate that the genetic ablation of this regulation via CRISPR/Cas9^26^ affects both the electrical and contractile properties of the heart, demonstrating that NCX1 Na^+^-dependent inactivation is important for proper heart function. These results are remarkable considering that they were gathered under normal physiological settings, without stressing the heart.

### NCX1 Na^+^-dependent inactivation shapes the electrical properties of the heart

NCX1 currents contribute to the plateau phase of the action potential^36,48,49^ and transgenic mice overexpressing NCX1 exhibit action potentials of longer duration^33,35,36,50^. Consistently, both electrocardiography investigations (Fig. 3) and patch-clamp studies in adult ventricular cells (Fig. 4) revealed significantly prolonged QT intervals and single-cell action potentials in K229Q mice, respectively. Several lines of experimental evidence support the hypothesis that the enhanced Na^+^ influx via NCX1 due to the removal of the Na^+^-dependent inactivation delays membrane potential repolarization. First, the NCX1 current in K229Q myocytes is significantly higher only when measured under conditions that promote Na^+^-dependent inactivation (*i.e*. high intracellular Na^+^, Fig. 5d–f). Second, we found no changes in NCX1 expression levels or trafficking patterns in K229Q myocytes (Fig. 6 and Supplementary Fig. 2). Third, the L-type Ca^2+^ current was unchanged in K229Q myocytes, excluding this as the underlying cause of the prolonged action potential (Fig. 7). Finally, we did not observe changes in mRNA levels of K_V_4.2 channels (Supplementary Fig. 2), an essential component of the I_to_ current previously linked to shortened action potential duration in NCX1 KO animals^3,33,51^. Similarly, the transcript levels of CaMKII, a kinase that targets a wide pool of EC coupling proteins^52,53^, were unchanged (Supplementary Fig. 2).

Together these findings support the hypothesis that the Na^+^-dependent inactivation process prevents excessive action potential prolongation associated with the electrogenic properties of NCX1. In fact, increased NCX1 function has been implicated in the genesis of both early and delayed afterdepolarizations and alternans^50,54–62^ and perhaps the Na^+^-dependent inactivation is a conserved feedback mechanism to limit the potential proarrhythmic properties of NCX1. Consistent with this view, we observed that the absence of NCX1 Na^+^-dependent inactivation favored a proarrhythmic regime within K229Q myocytes as they displayed a significantly higher incidence of early afterdepolarizations and alternans (Fig. 4e–i). The delayed repolarization seen in the absence of Na^+^-dependent inactivation is likely to provide the electrical substrate for L-type Ca^2+^ channel reactivation, thus inducing EADs^63–65^. Moreover, the prolonged action potential duration persisted at a higher frequency (5 Hz) (Fig. 4h) causing a shortening of the diastolic interval and thus promoting action potential alternans.

### NCX1 Na^+^-dependent inactivation affects Ca^2+^ dynamics and contractility

K229Q mice manifest a significant reduction in ejection fraction (11%) (Fig. 3c) and cell shortening (23%) (Fig. 8g). These changes were not associated with a decrease in the SR Ca^2+^ content as the amplitude of field stimulated and caffeine-induced Ca^2+^ transients were comparable between WT and K229Q myocytes (Figs. 8d and 9c). However, we observed two significant deviations in myocytes expressing the mutant exchanger: (1) the Ca^2+^ transient decay phase is accelerated (Figs. 6f and 7d), and (2) diastolic Ca^2+^ is reduced when measured at pacing rates similar to the physiological mouse heart rate (Fig. 6c). Since K229Q myocytes have similar Ca^2+^ transient amplitudes as control cells but start from a lower diastolic Ca^2+^ content, they may not be able to reach systolic Ca^2+^ levels (and hence force of contraction)^38^ of normal myocytes. Thus, the decreased diastolic Ca^2+^ is likely to contribute to the observed reduction in ejection fraction in K229Q mice.

Overall, the pathophysiological changes described above are consistent with an enhanced Ca^2+^ extrusion mediated by the activity of NCX1 lacking Na^+^-dependent inactivation. This hypothesis is further supported by the data showing that WT and K229Q myocytes present comparable SR Ca^2+^ load (Fig. 9) and reuptake (Supplementary Fig. 3). While more investigations are required to completely exclude other adaptive mechanisms (including changes in myofilament properties and level of saturation of cytoplasmic Ca^2+^ buffers)^66,67^, our study offers compelling evidence that the enhanced activity of NCX1 due to the lack of the Na^+^-dependent inactivation is the main contributor to the altered Ca^2+^ dynamics seen in K229Q myocytes. This evidence highlights the important role played by NCX1 Na^+^-dependent inactivation, which contributes to the tuning of NCX1 activity to maintain normal Ca^2+^ dynamics.

In summary, this study settles the long-standing question of whether Na^+^-driven NCX1 inhibition occurs in vivo, revealing its role in heart function under physiological settings. The inactivation of NCX1 by intracellular Na^+^ creates a reservoir of exchangers, which are effectively ‘silent’ in a normal physiological setting, preventing excessive Ca^2+^ and Na^+^ movements. As Na^+^-dependent inactivation can be overridden by elevations in intracellular Ca^2+,^^6,8,15,16^, inactivated transporters can be rescued by high levels of Ca^2+^ (µM range), providing an extra emergency exit route for Ca^2+^ under stress conditions. The removal of this inhibitory process, as in K229Q mice, enhances the number of active exchangers in the cell leading to increased Na^+^ influx and Ca^2+^ efflux, thus resulting in action potential prolongation, decreased heart contractility, and enhanced susceptibility to EADs and alternans.

While we assessed the relevance of this regulation under normal settings, we expect the Na^+^-dependent inactivation to play an even more critical role under conditions associated with elevated Na^+^, such as during ischemia/reperfusion injury and heart failure^17,21,27^. The high Na^+^ levels seen in these disorders are thought to reverse NCX1 transport mode^68,69^, leading to Ca^2+^ influx and cell death. The inactivation of NCX1 by intracellular Na^+^ may constitute a mechanism to limit Ca^2+^ entry and cell damage.

Finally, our study highlights the need to incorporate NCX1 Na^+^-dependent inactivation into mathematical models of the cardiac action potential^44,45^ to better reflect its modes of operation in physiological conditions.

## Methods

### K229Q mouse line generation

CRISPR/Cas9^26^ was used to create a protein-coding substitution, replacing lysine with glutamine (K229Q), in the *Slc8a1* gene, which encodes for isoform 1 of NCX (NCX1). All the alternative splice products of NCX1 will carry the mutation, including NCX1.1, the only variant found in the heart^1,31^. Note that K229Q numbering does not include the cleaved signal peptide of 32 amino acids. The mouse line (K229Q) was created by the Transgenic Mouse Facility of the University of California, Irvine in a C57BL/6N background. Mice were then back-crossed onto the C57BL/6J background. WT C57BL/6J were purchased from Jackson Laboratory. Only mice homozygous for the K229Q mutation are reported in this study. Successful mutation and homozygosity were confirmed via sequencing (Fig. 1c).

NCX activity and expression has been shown to be regulated by estrogen^70^ and estradiol^71^. Moreover, sex differences have been noted in the spatial distribution of NCX within the heart^70,72^. For these reasons, only male mice were investigated in this study. WT and K229Q mice of 12–16 weeks (3 months) of age were used unless otherwise stated in the Methods and figure legends. Animals were housed in the UCLA Division of Laboratory Animal Medicine (DLAM) barrier facility under temperature/humidity controlled conditions with a standard 12 h light/12 h dark cycle and were given free access to standard chow and water. Animal health and welfare was monitored by UCLA DLAM veterinary care staff. All animals used in this study were confirmed as K229Q by genotyping by Transnetyx using the primers – Forward: CAGCTCTCCTGGAGTTGTGG; Reverse: TCCAAAACCAGAGCCCCATC.

All animal protocols described herein were approved by the University of California, Los Angeles School of Medicine Animal Research Committee and strictly conformed to the Guide for Care and Use of Laboratory Animals published by the United States National Institutes of Health.

### Giant patch recordings

Mutagenesis, RNA synthesis, and electrophysiology were performed^13,16,73,74^. cRNAs encoding for the mouse WT and K229Q Na^+^-Ca^2+^ exchangers (NCX1.1) were injected into *Xenopus laevis* oocytes. Oocytes were kept at 18 °C for 4–7 days prior to electrophysiological recordings. Inside-out giant patch recordings of outward NCX1.1 currents were performed using borosilicate glass pipettes of ~20–30 µm. Intracellular solutions were rapidly changed using a computer controlled 20-channel solution switcher. Measurements were obtained using the following solutions: Pipette solution (mM): 100 NMG (N-methylglucamine), 10 HEPES (4-(2-hydroxyethyl)−1-piperazineethanesulfonic acid), 20 TEAOH (tetraethyl-ammonium hydroxide), 0.2 niflumic acid, 0.2 ouabain, 8 Ca(OH)_2_, pH = 7 (adjusted with methanesulfonic acid); Bath solution (mM): 100 CsOH or 100 NaOH, 20 TEAOH, 10 HEPES, 10 EGTA or HEDTA (N-(2-Hydroxyethyl) ethylenediamine-N,N’,N’-triacetic acid) and different Ca(OH)_2_ concentrations to obtain the desired final free Ca^2+^ concentrations, pH = 7 (using methanesulfonic acid). Free Ca^2+^ concentrations were calculated according to the WEBMAXC program and confirmed with a Ca^2+^ electrode (Kwik-Tip, WPI). Ca^2+^ concentrations (as determined by the Ca^2+^ electrode) are reported under the current traces (Fig. 1b). Data were acquired online at 4 ms/point and filtered at 50 Hz using an 8-pole Bessel filter. Experiments were performed at 35 °C at a holding potential of 0 mV.

### Heart weight to tibia length/body weight

Male mice were weighed prior to sacrifice. Mice were injected with heparin (200 UI/kg) to prevent blood coagulation, deeply anesthetized with isoflurane (5%) (confirmed by abolished pain reflexes), and subjected to cervical dislocation prior to thoracotomy. Hearts were excised by aortic, pulmonary, and vena cava transection and weighed (wet weight, mg). Subsequently, left tibias were excised and measured with digital calipers (mm).

### Echocardiography and electrocardiography

Male mice were lightly anesthetized with 1.5%–2.0% vaporized isoflurane with supplemental oxygen and placed on a 37 °C warming platform. B-mode and M-mode transthoracic echocardiography was performed using a VisualSonics Vevo 2100 with a 30 MHz linear transducer to analyze the cardiac hemodynamic parameters and heart structure of WT and K229Q homozygous mice. Left ventricular ejection fraction (%), left ventricular fractional shortening (%), left ventricular wall thicknesses (mm), left ventricular cavity volumes (μL), left ventricular cavity dimensions (mm), and septum thickness (mm) were determined using M-mode in Vevo LAB (5.5.1). Echocardiography was performed on mice at the following ages: 12–16 weeks (3 months), 24–28 weeks (6 months), 36–40 weeks (9 months), 50–54 weeks (12 months).

During the transthoracic echocardiography procedure, paws were secured to surface ECG leads and 5–10 s recordings were periodically collected in conjunction with B-mode and M-mode recordings. 15–20 ECG traces were measured from 2 to 4 recording periods at the stable heart rate of each animal. RR and QT intervals were measured using Trace Watcher III. QT intervals were corrected to QTc using Bazett’s correction to account for changes in heart rate^75^. Electrocardiography was performed on mice at 12–16 weeks old (3 months).

### Histological staining

Whole hearts of male WT and K229Q homozygous 12–16 week old (3 months) and 50–54 week old (12 months) mice were rinsed in Ca^2+^-free Tyrode’s solution and fixed overnight in 4% paraformaldehyde. Fixed hearts were paraffin-embedded and sectioned at 5 μm with a cryostat and then used for either hematoxylin and eosin staining (H&E) or Masson’s trichrome staining. Embedding, sectioning, and staining were conducted by the UCLA Translational Pathology Core Laboratory. Images were acquired at 10× magnification using a Motic Moticam 2500 attached to an Olympus IX71 microscope. To determine the amount of fibrosis, five images were obtained from each tissue section stained with Masson’s trichrome, which highlights collagen fiber in blue. Fibrosis was quantified as the fraction of blue staining to the total tissue slice area using Fiji ImageJ^76^.

### Isolation of adult ventricular cardiac myocytes

Ventricular cardiac myocytes were isolated from male WT and K229Q mice. Mice were injected with heparin (200 UI/kg) to prevent blood coagulation, deeply anesthetized with 5% isoflurane (confirmed by abolished pain reflexes), and subjected to cervical dislocation prior to thoracotomy. Hearts were quickly excised via aorta, pulmonary and vena cava transection, and cannulated (21 gauge) onto a gravity fed, constant-pressure Langendorff perfusion system (Radnoti). All perfusates were kept at 37 °C. Each heart was perfused with Ca^2+^-free Tyrode’s solution (mM: 136 NaCl, 5.4 KCl, 10 HEPES, 1.0 MgCl_2_, 0.33 NaH_2_PO_4_, 10 Glucose, pH 7.4) for ~5 min, followed by perfusion of Ca^2+^-free Tyrode’s enzyme solution containing collagenase (Type 2, 1 mg/ml, Worthington) and protease (Type XIV, 0.1 mg/ml, Sigma) for 10–13 min. The enzyme solution was washed out for ~8 min with Tyrode’s solution with 0.1 mM CaCl_2_. After excision, ventricular tissue was placed in a petri dish containing 0.1 mM CaCl_2_ Tyrode’s solution and then gently shredded with forceps. Cardiac ventricular myocytes were separated from dead and contaminant cells via filtration through a 100 μm sterile, nylon mesh cell strainer (Fisher). Single cells were then reintroduced to 1.0 mM CaCl_2_ for 15 min and then stored for up to 5 h in 1.8 mM CaCl_2_ at room temperature.

### Single-cell action potential recordings from isolated cardiac myocytes

Ventricular myocytes were patch-clamped in the whole-cell configuration in current-clamp mode. Borosilicate pipettes (TW150F‐3, World Precision Instruments) with a resistance of 1–2 MΩ were filled with an intracellular solution containing (mM): 130 KCl, 10 NaCl, 0.1 EGTA, 5 MgATP, 5 phosphocreatine, 10 HEPES, pH 7.2. The extracellular solution contained (mM): 136 NaCl, 5.4 KCl, 10 HEPES, 1 MgCl_2,_ 0.33 NaH_2_PO_4_, 10 Glucose, and 1 CaCl_2_, pH 7.4. Action potentials were elicited by supra-threshold 2 ms depolarizing pulses delivered at different pacing frequencies. All current-clamp experiments were performed at room temperature using an Axopatch 200B amplifier (Axon Instruments) and acquired using custom-made software (G-Patch). Signals were filtered at 1/5 of the sampling frequency. The action potential durations (APD), measured at 30%, 75%, and 90% of repolarization (APD_30_, APD_75_, APD_90_), were reported as an average of seven consecutive action potentials.

### NCX1 current recordings from isolated cardiac myocytes

Ventricular myocytes were patch-clamped in whole-cell configuration in voltage-clamp mode. The extracellular solution contained (mM): 130 NaMES, 10 CsCl, 0.33 NaH_2_PO_4_, 1 MgCl_2_, 10 HEPES, 1.8 CaCl_2_, 10 Glucose, 0.03 niflumic acid, 0.01 tetrodotoxin, 0.02 nifedipine, pH 7.4. Two different internal solutions were used in order to prevent or induce the Na^+^-dependent inactivation, respectively. To record NCX1 current in the absence of Na^+^-dependent inactivation, we used an intracellular solution lacking Na^+^ (Pipette solution, mM: 95 TEAMES, 7.2 TEACl, 25 CsMES, 7 CsBAPTA, 1 MgCl_2_, 10 HEPES, 5 MgATP, 3.2 CaCl_2_, 0.3 LiGTP, pH 7.2, 400 nM free Ca^2+^ as determined by a Ca^2+^ electrode). Na^+^-dependent inactivation in intact myocytes was promoted by replacing 25 mM CsMES with 25 mM NaMES (Pipette solution mM: 95 TEAMES, 7.2 TEACl, 25 NaMES, 10 CsBAPTA, 1 MgCl_2_, 10 HEPES, 5 MgATP, 3.2 CaCl_2_, 0.3 LiGTP, pH 7.2, 600 nM free Ca^2+^ as determined by a Ca^2+^ electrode). In this condition, 0.05 mM of ouabain and 0.005 mM ryanodine were added to the extracellular solution to help maintain constant intracellular Na^+^ and Ca^2+^ levels. Borosilicate pipettes (TW150F‐3, World Precision Instruments) with a resistance of 1–2 MΩ were used for the recordings.

Prior to recording NCX1 currents, cells were dialyzed for 5 min with the pipette solution. NCX1 currents were elicited using a ramp protocol from +80 mV to −100 mV (100 mV/s), starting from a holding potential of −40 mV. The protocol was repeated every 15 s and the average of 3 ramps was used for each experiment. At the end of each experiment, 10 mM NiCl_2_ was added to the extracellular solution. The resulting current was subtracted from the total current to obtain NCX1 nickel-sensitive currents. Current amplitudes were then normalized to cell capacitance. Recordings were performed at 35 °C. Data were acquired and analyzed using custom-made software (G-Patch, Analysis). Signals were filtered at 1/5 of the sampling frequency.

### L-type Ca^2+^ current recordings from isolated cardiac myocytes

Ventricular myocytes were patch-clamped in whole-cell configuration in voltage-clamp mode. Borosilicate pipettes were filled with an intracellular solution containing (mM): 110 Cs aspartate, 30 CsCl, 5 NaCl, 10 HEPES, 5 phosphocreatine, 5 MgATP, 1 EGTA, 0.05 cAMP, pH 7.2. The extracellular solution contained (mM): 136 NaCl, 5.4 CsCl, 0.33 NaH_2_PO_4_, 1 MgCl_2_, 10 HEPES, 10 Glucose, 1 CaCl_2_, 0.01 tetrodotoxin, pH 7.4. I_Ca,L_ was isolated by subtracting nifedipine-resistant current (0.02 mM nifedipine added to extracellular solution) from the total current. The voltage command protocol consisted of a 50 ms prepulse to −40 mV, followed by 100 ms pulses ranging from −40 to +80 mV in 10 mV increments, and returning to the holding potential of −80 mV. Recordings were performed at 35 °C and acquired and analyzed using custom-made software (G-Patch, Analysis). Signals were filtered at 1/5 of the sampling frequency.

### Immunocytochemistry

Adult ventricular cardiac myocytes from male mice were fixed in 2% paraformaldehyde in 0.1 M cacodylate buffer for 15 min at room temperature. Cells were blocked in PBS containing BSA (3%), Goat Serum (5%), and Triton X-100 (0.1%) for 1 h at room temperature, and incubated overnight with the mouse monoclonal antibody R3F1 to the cardiac NCX1 (1:50, a kind gift of Dr. K.D. Philipson, available from Swant)^32^ and rabbit monoclonal antibody to alpha-actinin 2 (1:200, Invitrogen, 7H1L79, Cat. No. 701914, Lot No. 2251595) at 4 °C. After washing for 3 × 10 min in PBST (PBS + 0.1% Tween 20, Sigma) cells were then incubated with goat anti-mouse Alexa Fluor 488 (1:200, Abcam, Cat. No. ab150113, Lot No. G33284150-1) and goat anti-rabbit Alexa Fluor 594 (1:200, Abcam, Cat. No. ab150080, Lot No. GR3323881-1) for 1 h at room temperature. Cells were then washed in PBST (3 × 10 min) and mounted (ProLong Gold, Invitorgen) to glass microscope slides. Images were taken via laser-scanning confocal microscopy (Nikon A1) using the same settings for WT and K229Q samples. Fluorescence images were collected with a 40× objective. Ventricular myocytes were analyzed one at a time by measuring their area and integrated density using Fiji ImageJ^76^. For each image, the mean background fluorescence was also determined. The corrected total cell fluorescence was then measured as CTCF = Integrated density of cell – (area of cell × mean background fluorescence)^77^.

### Immunoblotting

Heart ventricular tissue (~50 mg) was isolated from male WT and K229Q mice and homogenized on ice using Qiagen TissueRuptor in RIPA buffer (500 μl) with a protease inhibitor cocktail (Roche cOmplete). Each homogenate was centrifuged at 16,000 × *g* for 20 min at 4 °C. The supernatant was collected and protein concentration was determined via Pierce^TM^ BCA assay kit (ThermoFisher). Each lane represents a single heart homogenate with a total of ten ventricles analyzed for each group. Each sample was treated with SDS/beta-mercaptoethanol loading buffer at room temperature and then loaded at a concentration of 15 µg total protein. Proteins were separated on 4–20% SDS-polyacrylamide gels (GenScript) and transferred to PVDF membranes (Bio-Rad). The blot was divided to probe for NCX1 and GAPDH. Both sections were concomitantly blocked with 5% non-fat dry milk in PBST for 1 h at room temperature and probed with mouse monoclonal anti-NCX1 (1:2000, R3F1), or rabbit polyclonal anti-GAPDH (1:4000, Cell Signaling Technology, 14C10, Cat. No. 14C10, Lot No. 10) overnight at 4 °C. Anti-mouse-HRP (1:10,000, Sigma, Cat. No. A3682, Lot. No. 12M4754), or anti-rabbit-HRP secondary antibodies (1:10,000, Sigma, Cat. No. A0545, Lot. No. 102M4823) were used to reveal protein bands via chemiluminescence (Immobilon Forte - Millipore). Images were captured with a Bio-Rad ChemiDoc XRS. Proteins extracted from WT and K229Q ventricles were always blotted in tandem. Protein levels were quantified after blot background subtraction using Fiji ImageJ^76^. Each lane of the NCX1 signal was normalized to its respective GAPDH signal. Uncropped scans of blots can be found in the Source Data file.

### Real-time quantitative polymerase chain reaction (RT-qPCR)

Adult ventricular cardiac myocytes were isolated from male WT and K229Q mice and homogenized on ice for 20 s in TRIZOL reagent (1 mL per 50–100 mg of myocytes) using Fisher Model CL-18 Ultrasonic Homogenizer. Samples were incubated for 5 min at room temperature and cell debris was removed via centrifugation. The supernatant was transferred to a new tube and 200 μL of chloroform was added per 1 mL of TRIZOL. Samples were vortexed for 15 s and incubated at room temperature for 3 min. Samples were centrifuged at 12,000 × *g* for 15 min at 4 °C. The aqueous phase was transferred to a new tube and mixed with 500 μL of isopropyl alcohol per 1 mL of TRIZOL used. Following a RT 10 min incubation, samples were centrifuged at 12,000 × *g* for 10 min at 4 °C. The supernatant was removed and RNA pellet was washed with 1 mL of 75% ethanol per 1 mL of TRIZOL used. Samples were mixed and centrifuged at 7500 × *g* for 5 min at 4 °C. Pellet was washed again and ethanol was removed. RNA pellet was air-dried for 5–10 min and resuspended in DEPC-treated water. Genomic DNA was removed using RNeasy gDNA Eliminator Spin Column (Qiagen). For cDNA synthesis, 0.5 μg of RNA was reverse transcribed using iScript Reverse Transcription Supermix (Bio-Rad) according to the manufacturer’s protocol. RT-qPCR was performed with a Bio-Rad CFX96 Touch Real-time PCR Detection System using iQ SYBR Green Supermix (Bio-Rad) according to the manufacturer’s protocol. RNA was isolated from seven separate WT and K229Q ventricular myocyte preparations. For each sample, RT-qPCR determination was carried out in triplicate and the averaged value was collected. The expression 2^-ΔCt^ corresponds to the ratio of each gene versus GAPDH. The primers used for this analysis are shown in Supplementary Table 1.

### Ca^2+^ transients and cell shortening measurements from isolated cardiac myocytes

Isolated cardiac myocytes were incubated with 10 μM Fluo-4 AM (Invitrogen) and 0.1% Pluronic F-127 (Sigma) dissolved in Tyrode’s solution (mM: 136 NaCl, 5.4 KCl, 10 HEPES, 1.0 MgCl_2_, 0.33 NaH_2_PO_4_, 10 Glucose, 1.8 CaCl_2_, pH 7.4) for ~15 min at room temperature and mixed half-way through incubation period. Cells were then washed and placed in a 35 °C heated chamber with field stimulation (Warner Instruments). Prior to recordings, cells were given time to reach chamber temperature and then field stimulated via a 6 ms square pulse of constant voltage (10 V) using a MyoPacer Field Stimulator (IonOptix). To equilibrate the Ca^2+^ content in the sarcoplasmic reticulum, cells were paced at 1 Hz for 60 s before each recording. Cells were imaged using an inverted microscope (Zeiss Axiovert 135) equipped with a filter set (Chroma) comprised of an excitation filter (HQ480/40), emission filter (HQ525/50), and dichroic mirror (Q505lp). A 490 nm LED (Thorlabs) was used as a light source. Images were acquired with a 10× lens using either a sCMOS (Photometrics Cascade 128) or EMCCD cameras (Teledyne Princeton Instruments ProEM), operating at 50–100 frames/s with a pixel resolution of 512 × 512 (binned to 256 × 256). LightField (Teledyne Princeton Instruments) was used to capture camera images. Potential leak and bleaching of dye were determined by pacing the myocytes at 0.2 Hz at the end of the experiment allowing comparison with the initial recordings. Traces showing a significant decrease in fluorescence were excluded from the analysis. After subtraction of nonspecific background fluorescence, the signal was measured from an identified region of interest (ROIs) within each single myocyte that encompassed ~80%–90% of its total surface in the contracted state. The changes in diastolic baseline fluorescence at different pacing frequencies were calculated as (F − F_0@0.2Hz_)/F_0@0.2Hz_ where F is the diastolic baseline fluorescence at 0.2, 1, 2, and 5 Hz and F_0@0.2Hz_ is the baseline fluorescence at 0.2 Hz. The amplitude of the Ca^2+^ transients recorded at different pacing rates was instead determined as (F_Peak_ – F_Baseline_)/F_0@0.2Hz_, where F_Peak_ and F_Baseline_ are the peak and baseline fluorescence at each frequency and F_0@0.2Hz_ is the baseline fluorescence measured at 0.2 Hz. Finally, Ca^2+^ transients were fit with a single exponential to extrapolate the time constant of decay (τ values) while the Ca^2+^ transient duration was calculated at half maximum amplitude.

For caffeine-induced Ca^2+^ transient measurements, cells were placed in a 35 °C heated chamber onto glass coverslips coated with CellTak (1:100 in 0.1 M NaHCO_3_). Cells were then field stimulated for 60 s at 1 Hz to equilibrate SR Ca^2+^ loading. Afterwards, control transients were recorded by pacing the cells at 1 Hz for 15 s. Pacing was then stopped for 4 s, and 20 mM caffeine was rapidly perfused (Picospritzer II, Parker Hannifin). The amplitude of the caffeine transient was calculated as (F − F_0_)/F_0_ where F is the peak during caffeine puff and F_0_ is the resting fluorescence. The decay of the caffeine-induced Ca^2+^ transient was fit to a single exponential to extrapolate the time constant (τ values).

SERCA activity was measured following a protocol described by Bassani et al.^39^. Adult ventricular myocytes were placed onto glass coverslips coated with CellTak then transferred to a 30 °C heated chamber. Cells were field stimulated for 120 s at 0.5 Hz to equilibrate SR Ca^2+^ loading, control transients were then recorded for 30 s while perfused with Tyrode’s solution (mM: 140 NaCl, 6 KCl, 1 MgCl_2_, 1 CaCl_2_, 10 Glucose, 5 HEPES, pH 7.4 with NaOH, 30 °C). Field stimulation was paused and cells were bathed for 9 min with a modified Tyrode’s solution lacking both Na^+^ and Ca^2+^ (mM: 140 LiCl, 6 KCl, 1 MgCl_2_, 1 EGTA, 10 Glucose, 5 HEPES, pH 7.4 with LiOH, 30 °C). This process depletes the cells of intracellular Na^+^ ^39^. Ca^2+^ was then reintroduced by perfusing a solution containing 1 mM Ca^2+^ but lacking extracellular Na^+^ (mM: 140 LiCl, 6 KCl, 1 MgCl_2_, 1 CaCl_2_, 10 Glucose, 5 HEPES, pH 7.4 with LiOH, 30 °C). The pacing was reinstated after a complete exchange of solution (~45 s). The decay time constant was measured by fitting the Ca^2+^ transient relaxation to a single exponential. Only cells devoid of aberrant Ca^2+^ oscillations and waves both before and after intracellular/extracellular Na^+^ removal were included in the analysis. Approximately ~12% of cells studied survived the described experimental procedure (~88% of cells displayed Ca^2+^ oscillations or waves upon reintroduction of 1 mM Ca^2+^ following Na^+^ depletion).

Cell shortening was measured from bright-field recordings of isolated ventricular myocytes field stimulated at 1 Hz. Resting and contracted cell lengths were measured in Fiji ImageJ^76^ and represented as the percentage of stimulated shortening (stimulated) relative to the resting cell length (unstimulated).

### Statistical analyses

All *P* values were calculated using an unpaired, two-tailed Welch’s *t*-test, except for a Fisher’s exact test used for comparing data in Fig. 8i and a paired, two-tailed ratio *t*-test used for comparing paired cells in Fig. 9c. Normal sample distributions were assumed. *P* values are represented as follows: ^****^P < 0.0001; ^***^P < 0.001; ^**^P < 0.01; ^*^P < 0.05. Specific *P* values are included in the Source Data file if not stated in the figure legends. Statistical analyses were performed with Prism 9 (GraphPad). Analyses in which *P* > 0.05 are unmarked. Data are represented as mean ± SEM, unless otherwise stated. Experimental ‘n’ is noted throughout the text and distinguishes between the number of cells and animals used.

### Reporting summary

Further information on research design is available in the Nature Portfolio Reporting Summary linked to this article.

### Supplementary information

Supplementary Information

Reporting Summary

### Source data

Source data

## Data Availability

All data are included in the main manuscript and related supplementary files. The structure of human NCX1 used in Fig. 1a was obtained from the Protein Data Bank (PDB: 8SGJ)^28^ and visualized using PyMOL. Source data are provided with this paper.
