# Supplementary material for: Cardiac function is regulated by the sodium-dependent inhibition of the sodium-calcium exchanger NCX1
Source: Nat Commun. 2024 May 7;15:3831. doi: 10.1038/s41467-024-47850-z (PMC11076594; doi:10.1038/s41467-024-47850-z)
Supplement: Supplementary file 1 — Supplementary Information [file 41467_2024_47850_MOESM1_ESM.pdf]

Supplementary Information

Cardiac function is regulated by the sodium-dependent inhibition of the sodium-calcium exchanger NCX1

Scranton et al.

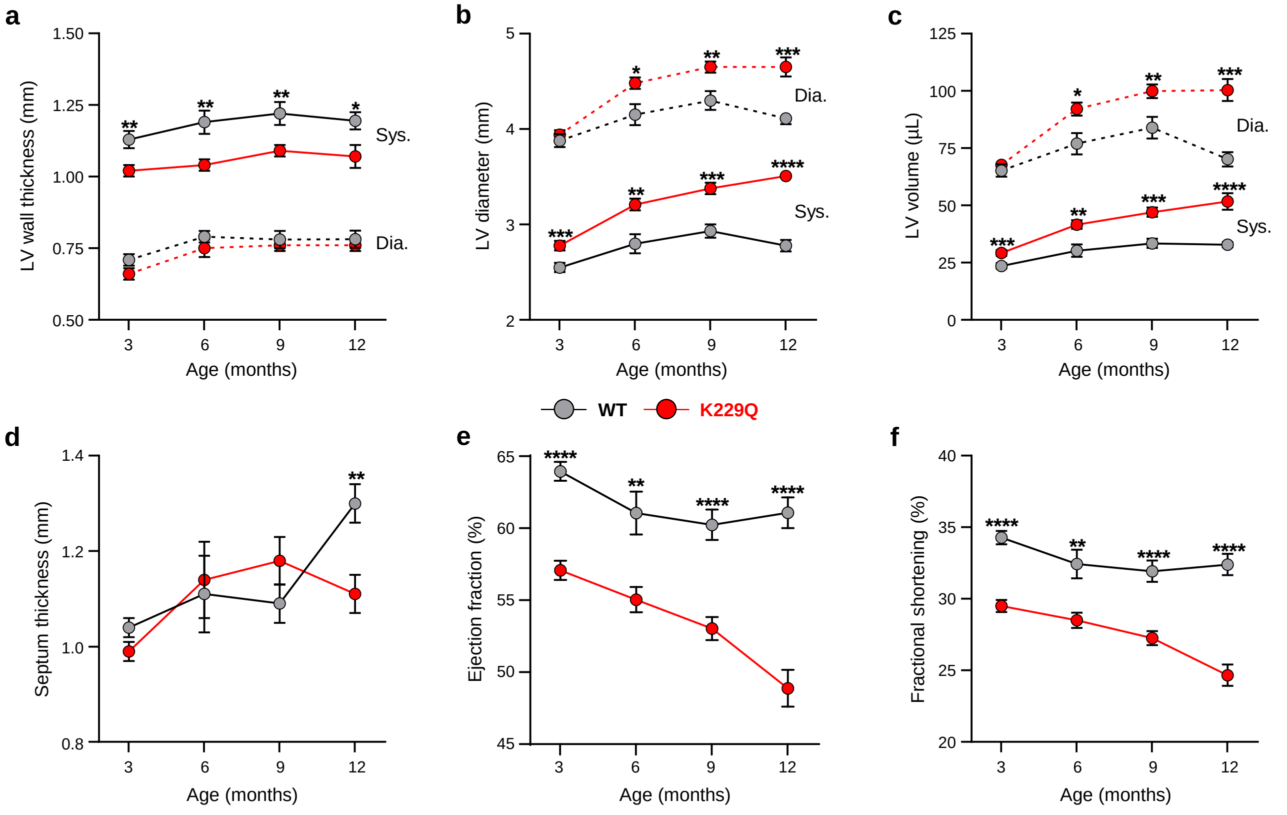

Supplementary Figure 1: Echocardiography in K229Q mice indicates sustained changes in cardiac function and morphology with age. WT (grey) and K229Q (red) mice were evaluated by echocardiography under light anesthesia at 3, 6, 9, and 12 months of age. **a**) Left ventricular (LV) wall thickness (mm). **b**) LV diameter (mm). **c**) LV diastolic (Dia.) and systolic (Sys.) volume (μL). **d**) Septum thickness (mm). **e**) LV ejection fraction (%). **f**) LV fractional shortening (%). Impairment of cardiac contractility and alteration in heart dimensions between WT and K229Q mice is sustained with age (12-16 weeks, 3 months: WT n = 15; K229Q n = 17) (24-28 weeks, 6 months: WT n = 10; K229Q n = 8) (36-40 weeks, 9 months: WT n = 12; K229Q n = 11) (50-54 weeks, 12 months: WT n = 13; K229Q n = 11). Data reported as the mean ± SEM (two-tailed Welch's t-test; *****P* < 0.0001, ****P* < 0.001, ***P* < 0.01, **P* < 0.05). Specific *P* values and source data are provided in the Source Data file.

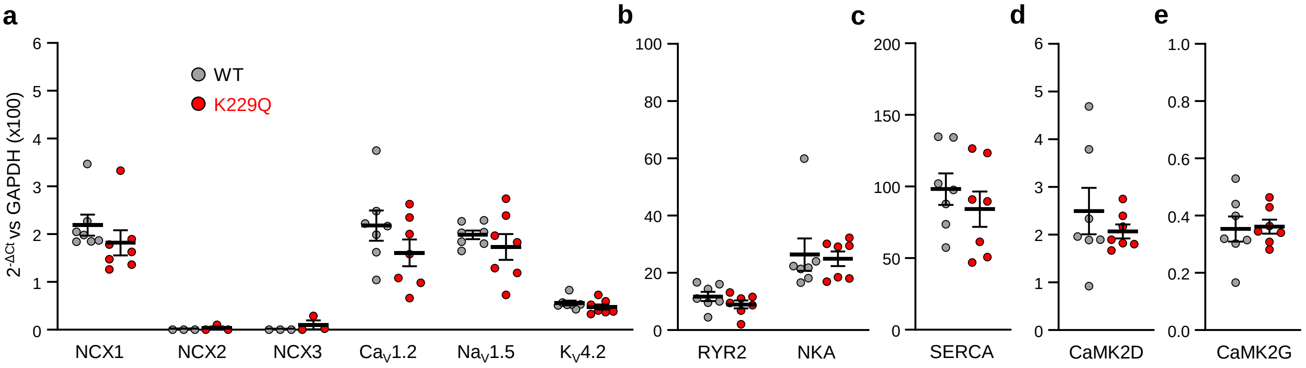

Supplementary Figure 2: The levels of mRNA encoding for proteins essential for excitation-contraction coupling are not altered in myocytes expressing K229Q exchanger.

Shown are the transcript levels of sampled EC coupling proteins. mRNA was extracted from isolated adult ventricular cardiac myocytes. Values are expressed as $2^{-\Delta Ct}$ relative to GAPDH (WT, grey; K229Q, red; 7 animals per group). Transcripts levels of NCX2 and NCX3 were considered not detectable (3 animals per group). The removal of Na^+-dependent inactivation did not alter the expression of the genes sampled relevant to EC coupling, including NCX1. The proteins investigated were as follows (gene name provided in parenthesis): **a**) NCX1 (*Slc8a1*); NCX2 (*Slc8al2*); NCX3 (*Slc8al3*); $Ca_V1.2$ (*Cacna1c*); $Na_V1.5$ (*Scn5a*); $K_V4.2$ (*Kcnd2*). **b**) RYR2 (*Ryr2*); Na^+/K^+-ATPase (NKA) (*Atp1a1*). **c**) Sarco/endoplasmic Ca^{2+}-ATPase (SERCA) (*Atp2a2*). **d**) CaMK2D (*Camk2d)*. **e**) CaMK2G (*Camk2g)*. Data represented as mean ± SEM (two-tailed Welch's t-test). Source data are provided in the Source Data file.

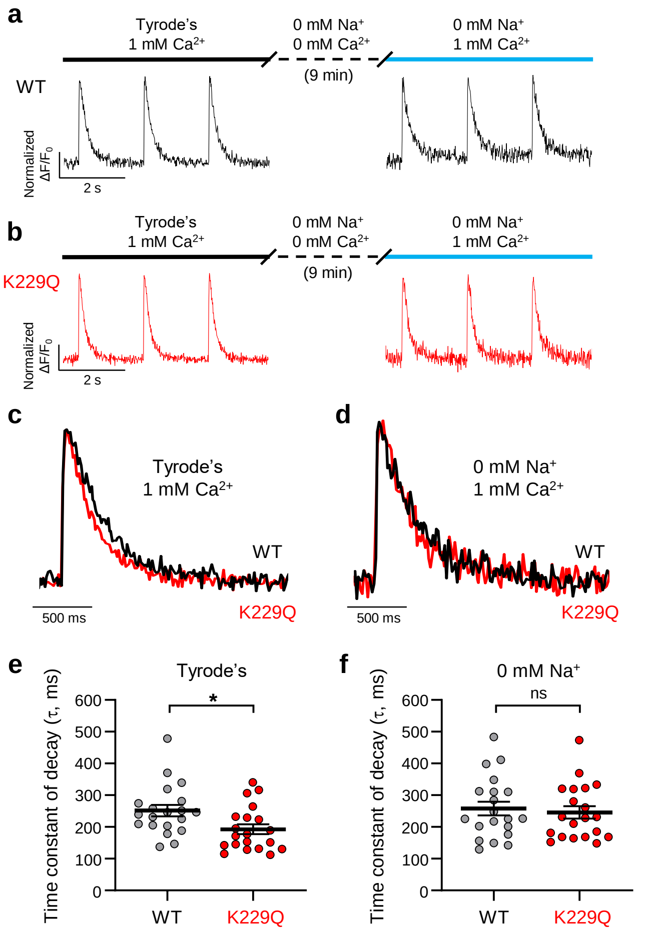

Supplementary Figure 3: SERCA activity is unchanged in K229Q myocytes.

a, b) Representative normalized Ca^{2+} transients recorded from field stimulated WT (**a,** black) and K229Q (**b,** red) ventricular myocytes. Ca^{2+} transients were elicited by pacing myocytes at 0.5 Hz at 30 ºC. After acquisition of Ca^{2+} transients in control conditions (Tyrode's, 1 mM Ca^{2+}), pacing was paused and cells were incubated for 9 minutes in a solution lacking both extracellular Na^+ (replaced with 140 mM Li^+) and Ca^{2+}. This procedure depletes cells of intracellular Na^+. Ca^{2+} was then reintroduced in the presence of 140 mM Li^+ (0 mM Na^+, 1 mM Ca^{2+}; blue line above trace). As Li^+ is not transported by NCX, the exchanger is inactive and the decay of the transients is driven by SERCA. **c, d**) WT (black) and K229Q (red) Ca^{2+} transients, recorded in the indicated conditions, were scaled and superimposed. **e, f**) Decay time constant (τ, ms) values obtained from Ca^{2+} transients recorded in control conditions (Tyrode's solution) (**e**) and in the absence of NCX1 activity (0 mM Na^+, 1 mM Ca^{2+}) (**f**). Note that in control conditions the decay of the Ca^{2+} transient is faster in K229Q myocytes. Preventing NCX1 activity by removing its substrates abolishes this difference. WT, grey, n = 20/6 cells/animals; K229Q, red, n = 20/8 cells/animals. Data represented as mean ± SEM (two-tailed Welch's t-test; *P = 0.0175). Source data are provided in the Source Data file.

Protein	Gene	Primer (Forward)	Primer (Reverse)
CaMK2D	*Camk2d*	ATAGAAGTTCAAGGCGACCAG	CAGCAAGATGTAGAGGATGACG
CaMK2G	*Camk2g*	AAACCTGTGGATATCTGGGC	CTGGTGATGGGAAATCGTAGG
Ca_V1.2	*Cacna1c*	TCCCGAGCACATCCCTACTC	ACTGACGGTAGAGATGGTTGC
GAPDH	*Gapdh*	TCACCACCATGGAGAAGGC	GCTAAGCAGTTGGTGGTGCA
K_V4.2	*Kcnd2*	TGACAACACTGGGGTATGGC	CTGCTCGTTGGTTTTGGTGG
Na_V1.5	*Scn5a*	TCCGCCTTGGACCAAATATGT	CACACTGAAGTCTAGCCAGTT
Na^+/K^+-ATPase	*Atp1a1*	CTGGCTGAGAACGGTTTCCT	AACGCTGTATGGCAGGTGAA
NCX1	*Slc8a1*	CGCTGGGGAAGATGACGATG	TGGGACGAAGGCAAACAGAAC
NCX2	*Slc8a2*	ACCCTCGCAGTGGAATCATC	CTCAGTGCCCACGAATGTG
NCX3	*Slc8a3*	CCTGAATTGTCTTCGAGCAGAG	TCCCCAAGGGAAGGGTTCT
RYR2	*Ryr2*	AAAAGTGCGTGTTGGAGATGA	CACCGCCAATGAGATAGCCT
SERCA	*Atp2a2*	ATGGAGAACGCTCACACAAAG	ACTGCTCAATCACAAGTTCCA

Supplementary Table 1: List of primers used for RT-qPCR.
Forward and reverse primer sequences used with RT-qPCR. Ca^{2+}/calmodulin dependent protein kinase II delta (CaMK2D, *Camk2d*); Ca^{2+}/calmodulin dependent protein kinase II gamma (CaMK2G, *Camk2g*); L-type Ca^{2+} channel (Ca_V1.2, *Cacna1c*); Glyceraldehyde 3-phosphate dehydrogenase (GADPH; *Gapdh*); Voltage-dependent K^+ channel (K_V4.2, *Kcnd2*); Voltage-dependent Na^+ channel (Na_V1.5, *Scn5a*); Na^+/K^+-ATPase (NKA, *Atp1a1*); Na^+-Ca^{2+} exchangers (NCX1, *Slc8a1*; NCX2, *Slc8a2*; NCX3, *Slc8a3*); Ryanodine receptor (RYR2, *Ryr2*); Sarco/endoplasmic Ca^{2+}-ATPase (SERCA, *Atp2a2*).
