# Supplementary material for: Cardiac function is regulated by the sodium-dependent inhibition of the sodium-calcium exchanger NCX1
Source: Nat Commun. 2024 May 7;15:3831. doi: 10.1038/s41467-024-47850-z (PMC11076594; doi:10.1038/s41467-024-47850-z)
Supplement: Supplementary file 2 — Reporting Summary [file 41467_2024_47850_MOESM2_ESM.pdf]

nature portfolio

Corresponding author(s): Michela Ottolia

Last updated by author(s): Feb 26, 2024

Reporting Summary

Nature Portfolio wishes to improve the reproducibility of the work that we publish. This form provides structure for consistency and transparency in reporting. For further information on Nature Portfolio policies, see our Editorial Policies and the Editorial Policy Checklist.

Statistics

For all statistical analyses, confirm that the following items are present in the figure legend, table legend, main text, or Methods section.

n/a	Confirmed	
☐	☒	The exact sample size (*n*) for each experimental group/condition, given as a discrete number and unit of measurement
☐	☒	A statement on whether measurements were taken from distinct samples or whether the same sample was measured repeatedly
☐	☒	The statistical test(s) used AND whether they are one- or two-sided<br>*Only common tests should be described solely by name; describe more complex techniques in the Methods section.*
☒	☐	A description of all covariates tested
☒	☐	A description of any assumptions or corrections, such as tests of normality and adjustment for multiple comparisons
☐	☒	A full description of the statistical parameters including central tendency (e.g. means) or other basic estimates (e.g. regression coefficient) AND variation (e.g. standard deviation) or associated estimates of uncertainty (e.g. confidence intervals)
☐	☒	For null hypothesis testing, the test statistic (e.g. *F*, *t*, *r*) with confidence intervals, effect sizes, degrees of freedom and *P* value noted<br>*Give P values as exact values whenever suitable.*
☒	☐	For Bayesian analysis, information on the choice of priors and Markov chain Monte Carlo settings
☒	☐	For hierarchical and complex designs, identification of the appropriate level for tests and full reporting of outcomes
☒	☐	Estimates of effect sizes (e.g. Cohen's *d*, Pearson's *r*), indicating how they were calculated

Our web collection on statistics for biologists contains articles on many of the points above.

Software and code

Policy information about availability of computer code

Data collection	Calcium transients were recorded using either a sCMOS or EMCCD camera (Teledyne Princeton Instruments) captured using LightField (Teledyne Princeton Instruments).<br>Cardiac myocytes were imaged using an A1R HD25 confocal microscope with NIS-Elements (Nikon).<br>Echocardiography was performed using a VisualSonics Vevo 2100 with a 30 MHz linear transducer.<br>NCX1 currents, calcium currents, and action potentials were acquired using G-Patch.<br>RT-qPCR was performed with Bio-Rad CFX96 Touch Real-time PCR Detection System.
Data analysis	Calcium handling and action potential analysis was performed using ClampFit (pCLAMP 10).<br>ECG traces were analyzed using Trace Watcher III.<br>Echocardiography data was analyzed with Vevo LAB (5.5.1).<br>Fibrosis, Western blot, cell shortening, and CTCF analysis was performed using Fiji ImageJ.<br>NCX1 currents and calcium currents were analyzed using Analysis.<br>Statistical analyses were performed with Prism 9 (GraphPad).

For manuscripts utilizing custom algorithms or software that are central to the research but not yet described in published literature, software must be made available to editors and reviewers. We strongly encourage code deposition in a community repository (e.g. GitHub). See the Nature Portfolio guidelines for submitting code & software for further information.

Data

Policy information about availability of data

All manuscripts must include a data availability statement. This statement should provide the following information, where applicable:

- Accession codes, unique identifiers, or web links for publicly available datasets
- A description of any restrictions on data availability
- For clinical datasets or third party data, please ensure that the statement adheres to our policy

All data are included in the main manuscript and related supplementary files. The structure of human NCX1 used in Figure 1a was obtained from the Protein Data Bank (PDB: 8SGJ; https://doi.org/10.2210/pdb8SGJ/pdb) and visualized using PyMOL. Source data are provided with this paper.

Research involving human participants, their data, or biological material

Policy information about studies with human participants or human data. See also policy information about sex, gender (identity/presentation), and sexual orientation and race, ethnicity and racism.

Reporting on sex and gender	N/A
Reporting on race, ethnicity, or other socially relevant groupings	N/A
Population characteristics	N/A
Recruitment	N/A
Ethics oversight	N/A

Note that full information on the approval of the study protocol must also be provided in the manuscript.

Field-specific reporting

Please select the one below that is the best fit for your research. If you are not sure, read the appropriate sections before making your selection.

☒ Life sciences ☐ Behavioural & social sciences ☐ Ecological, evolutionary & environmental sciences

For a reference copy of the document with all sections, see nature.com/documents/nr-reporting-summary-flat.pdf

Life sciences study design

All studies must disclose on these points even when the disclosure is negative.

Sample size	Sample size was determined with reference to similar sample sizes used in previously published work on NCX1 in mouse models. For electrocardiography and echocardiography, a sample size of 15 animals per group gives adequate power (80%) to reliably detect standardized effect sizes as small as 1.06 between groups (two-sample t-test, two-tailed, alpha=0.05). For electrophysiological studies, patch clamp is a notoriously difficult technique, which leads to a limited sample size. Accordingly, successful publications employing these approaches report standard error values calculated from 6 to 12 cells (Torrente et al., J Phy, 2017; Moroni et al., Nat Comm, 2024). Additionally, adequate sample size was determined based on the consistency and magnitude of differences measured between the WT and K229Q mice.
Data exclusions	Action potential duration measured from one K229Q cell was not included in APD measurements (Fig. 6) due to extreme prolongation of action potential duration. However, this cell was included in "Fraction of aberrant action potentials" (Fig. 6i). Otherwise, data were not excluded.
Replication	All experiments were performed in multiple animals, and in multiple cells per animals when applicable, to ensure reproducibility of experimental findings. All attempts at replication were successful. When possible, experimental investigation of WT and K229Q animals were done simultaneously, or on the same day. All experimental animals were age and sex-matched. For each experiment, at least 3 independent replicates were performed for each genotype.
Randomization	The requirement for animals to have a specific genetic signature did not allow for randomization. When possible, experimental investigation of WT and K229Q animals were done simultaneously, or on the same day. All experimental animals were age and sex-matched.
Blinding	Investigators were not blinded to mouse genotype during experiments as it was necessary to ensure WT and K229Q experiments were performed simultaneously, or on the same day. In addition, genotyping/caring for the colony and performing experiments and analysis were performed by the same person, not allowing for blinding to occur.

Reporting for specific materials, systems and methods

We require information from authors about some types of materials, experimental systems and methods used in many studies. Here, indicate whether each material, system or method listed is relevant to your study. If you are not sure if a list item applies to your research, read the appropriate section before selecting a response.

Materials & experimental systems

n/a	Involved in the study
☐	☒ Antibodies
☒	☐ Eukaryotic cell lines
☒	☐ Palaeontology and archaeology
☐	☒ Animals and other organisms
☒	☐ Clinical data
☒	☐ Dual use research of concern
☒	☐ Plants

Methods

n/a	Involved in the study
☒	☐ ChIP-seq
☒	☐ Flow cytometry
☒	☐ MRI-based neuroimaging

Antibodies

Antibodies used

anti-NCX1, R3F1 (1:50 ICC dilution/1:2000 Western dilution, Received as a gift from Dr. KD Philipson - Available commercially from Swant)
anti-alpha-actinin 2, 7H1L79 (1:200 dilution, Invitrogen, Cat. No. 701914, Lot No. 2251595)
anti-GAPDH, 14C10 (1:4000 dilution, Cell Signaling Technology, Cat. No. 14C10, Lot No. 10)
goat anti-mouse horseradish peroxidase (HRP)-conjugated secondary (1:10000 dilution, Sigma, Cat. No. A3682, Lot. No. 12M4754)
goat anti-rabbit horseradish peroxidase (HRP)-conjugated secondary (1:10000 dilution, Sigma, Cat. No. A0545, Lot. No. 102M4823)
goat anti-mouse Alexa Fluor 488 (1:200 dilution, Abcam, Cat. No. ab150113, Lot No. G33284150-1)
goat anti-rabbit Alexa Fluor 594 (1:200 dilution, Abcam, Cat. No. ab150080, Lot No. GR3323881-1)

Validation

Antibodies have been validated by the respective vendors for the applications described in the manuscript. R3F1 validation is provided commercially (www.swant.com) and has been published by Dr. KD Philipson (Porzig et al., Am. J. Physiol. 1993). Anti-NCX1, anti-alpha-actinin 2, anti-GAPDH, anti-mouse HRP, anti-rabbit HRP, anti-mouse Alex 488, and anti-rabbit Alex 594 were validated for use in mice for the applications used in the manuscript by their respective manufacturers:

anti-alpha-actinin 2, 7H1L79 (https://www.thermofisher.com/antibody/product/alpha-Actinin-2-Antibody-clone-7H1L69-Recombinant-Monoclonal/701914)
anti-GAPDH, 14C10 (https://www.cellsignal.com/products/primary-antibodies/gapdh-14c10-rabbit-mab/2118
anti-mouse HRP (https://www.sigmaaldrich.com/US/en/product/sigma/a3682)
anti-rabbit HRP (https://www.sigmaaldrich.com/US/en/product/sigma/a0545)
anti-mouse Alexa 488 (https://www.abcam.com/products/secondary-antibodies/goat-mouse-igg-hl-alexa-fluor-488-ab150113.html)
anti-rabbit Alexa 594 (https://www.abcam.com/products/secondary-antibodies/goat-rabbit-igg-hl-alexa-fluor-594-ab150080.html)

Animals and other research organisms

Policy information about studies involving animals; ARRIVE guidelines recommended for reporting animal research, and Sex and Gender in Research

Laboratory animals

The K229Q mouse line was created by the Transgenic Mouse Facility of the University of California, Irvine in a C57BL/6N background. Mice were then back-crossed onto the C57BL/6J background. WT C57BL/6J were purchased from Jackson Laboratory. Male mice were exclusively used in this study. Genotyping was confirmed by Transnetxy. Primary age of animals used was 12-16 weeks old (3 months) unless otherwise stated in Methods and figure legends. Briefly, histological staining was performed on animals aged 50-54 weeks old (12 months), and echocardiography was performed on animals aged 24-28 weeks old (6 months), 36-40 weeks old (9 months), and 50-54 weeks old (12 months). For euthanasia, animals were deeply anesthetized with isoflurane (confirmed by abolished pain reflexes) and subjected to cervical dislocation. Animals were housed at the UCLA Division of Laboratory Animal Medicine (DLAM) barrier facility under temperature/humidity controlled conditions with a standard 12 hour light/12 hour dark cycle and were given free access to standard chow and water. Animal health and welfare was monitored by UCLA DLAM veterinary care staff.

Wild animals

The study did not involve wild animals.

Reporting on sex

The reported investigation involved the study of only male wild-type and homozygous K229Q mice. While we plan to also investigate the impact of the removal of NCX1 Na+-dependent inactivation in female mice, we used solely male mice as NCX activity and expression has been shown to be regulated by estrogen (Chen et al., J Physiol, 589(5), 1061-1080, 2011) and estradiol (Sánchez et al., Cell Mol Neurobiol, 31, 619-627, 2011). Sex-differences have also been noted in the spatial distribution of NCX within the heart (Chen et al., J Physiol, 589(5), 1061-1080, 2011; Papp et al., Biol Sex Differ, 8, 26, 2017). For these reasons, we have tried to reduce the number of variables that may influence the findings reported in our initial manuscript describing this mouse line.

Field-collected samples

The study did not involve samples collected from the field.

Ethics oversight	All animals protocols were approved by the University of California, Los Angeles School of Medicine Animal Research Committee. This investigation strictly conformed to the Guide for Care and Use of Laboratory Animals published by the United States National Institutes of Health.

Note that full information on the approval of the study protocol must also be provided in the manuscript.
